# Supplementary figures and images for: Ush regulates hemocyte-specific gene expression, fatty acid metabolism and cell cycle progression and cooperates with dNuRD to orchestrate hematopoiesis
Source: PLoS Genet. 2021 Feb 18;17(2):e1009318. doi: 10.1371/journal.pgen.1009318 (PMC7891773; doi:10.1371/journal.pgen.1009318)

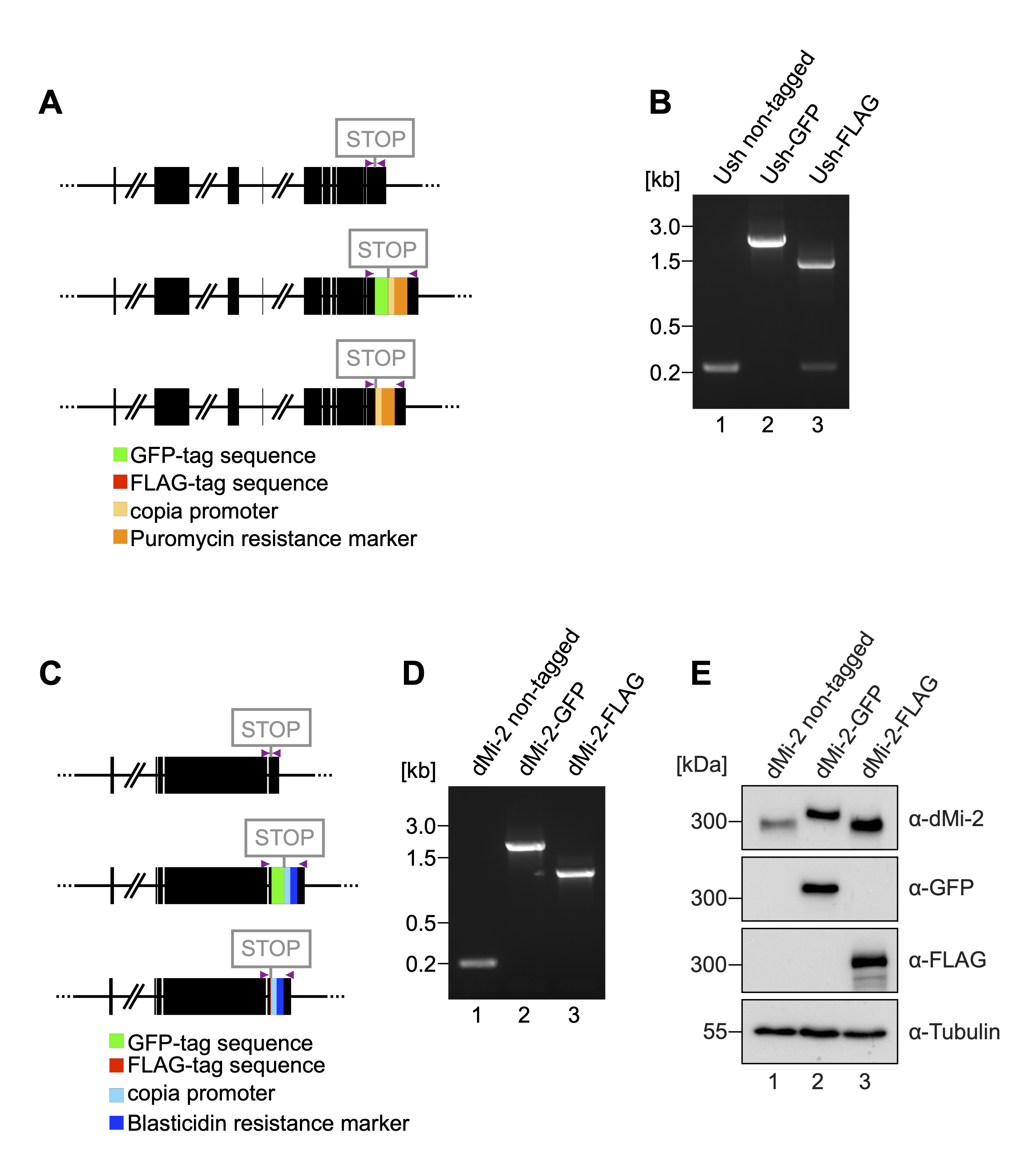

Supplement: S1 Fig — A Schematic representation of the Ush gene locus before (top) and after insertion of GFP (middle) and FLAG (bottom) tagging constructs. Black boxes represent exons, black (broken) lines represent introns. The inserted tag sequences (GFP: green, FLAG, red) and selection marker (promoter: ochre, Puromycin resistance: orange) are highlighted. The positions of primers used for genotyping of Ush alleles are indicated with purple arrowheads. B PCR from genomic DNA of control cells and cells modified to express GFP- or FLAG-tagged Ush, respectively. Insertion of the tag sequence followed by a Puromycin selection marker is monitored using primers surrounding the 3’ end of the coding region within the Ush gene. Non-tagged alleles give rise to a 216 bp amplicon, GFP- and FLAG-tagged alleles result in 1991 bp and 1311 bp fragments respectively. C Schematic representation of the dMi-2 gene locus before (top) and after insertion of GFP (middle) and FLAG (bottom) tagging constructs. Black boxes represent exons, black (broken) lines represent introns. The inserted tag sequences (GFP: green, FLAG, red) and selection marker (promoter: light blue, Blasticidin resistance: dark blue) are highlighted. The positions of primers used for genotyping of Ush alleles are indicated with purple arrowheads. D PCR from genomic DNA of control cells and cells modified to express GFP- or FLAG-tagged dMi-2, respectively. Insertion of the tag sequence followed by a Blasticidin selection marker is monitored using primers surrounding the 3’ end of the coding region within the Ush gene. Non-tagged alleles give rise to a 200 bp amplicon, GFP- and FLAG-tagged alleles result in 1737 bp and 1077 bp fragments respectively. E Nuclear extracts of control cells and cells expressing endogenously tagged dMi-2-GFP or dMi-2-FLAG was probed on Western blot using antibodies against dMi-2, GFP or FLAG. Tubulin signal serves as loading control. (TIF) [file pgen.1009318.s001.tif]

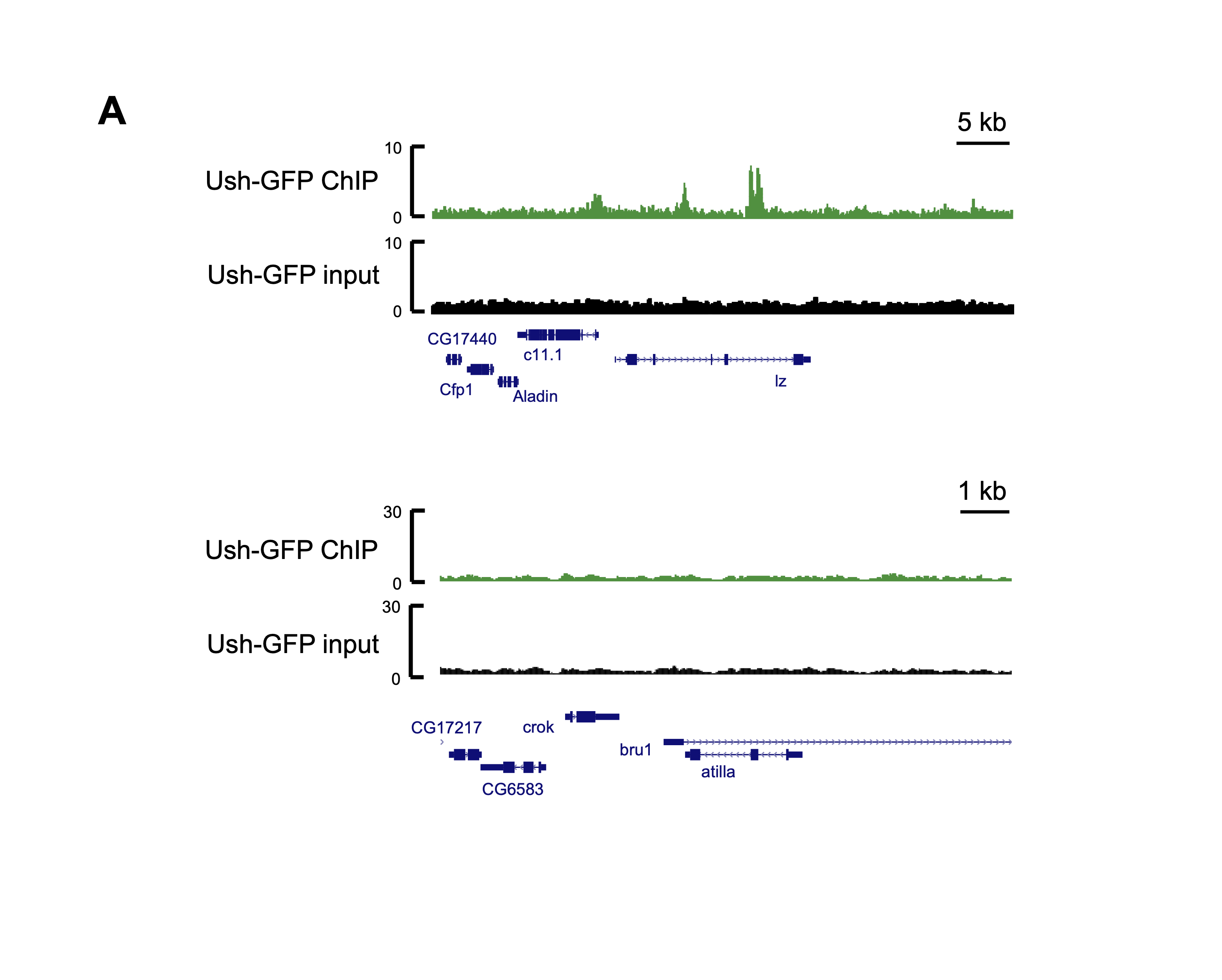

Supplement: S2 Fig — A Genome browser snapshots of the lozenge (lz) (top) and the atilla (bottom) gene locus displaying Ush occupancy (green) determined by Ush-GFP ChIP-seq. Input signals are shown in black. Location of genes is displayed below with boxes indicating exons. (TIF) [file pgen.1009318.s002.tif]

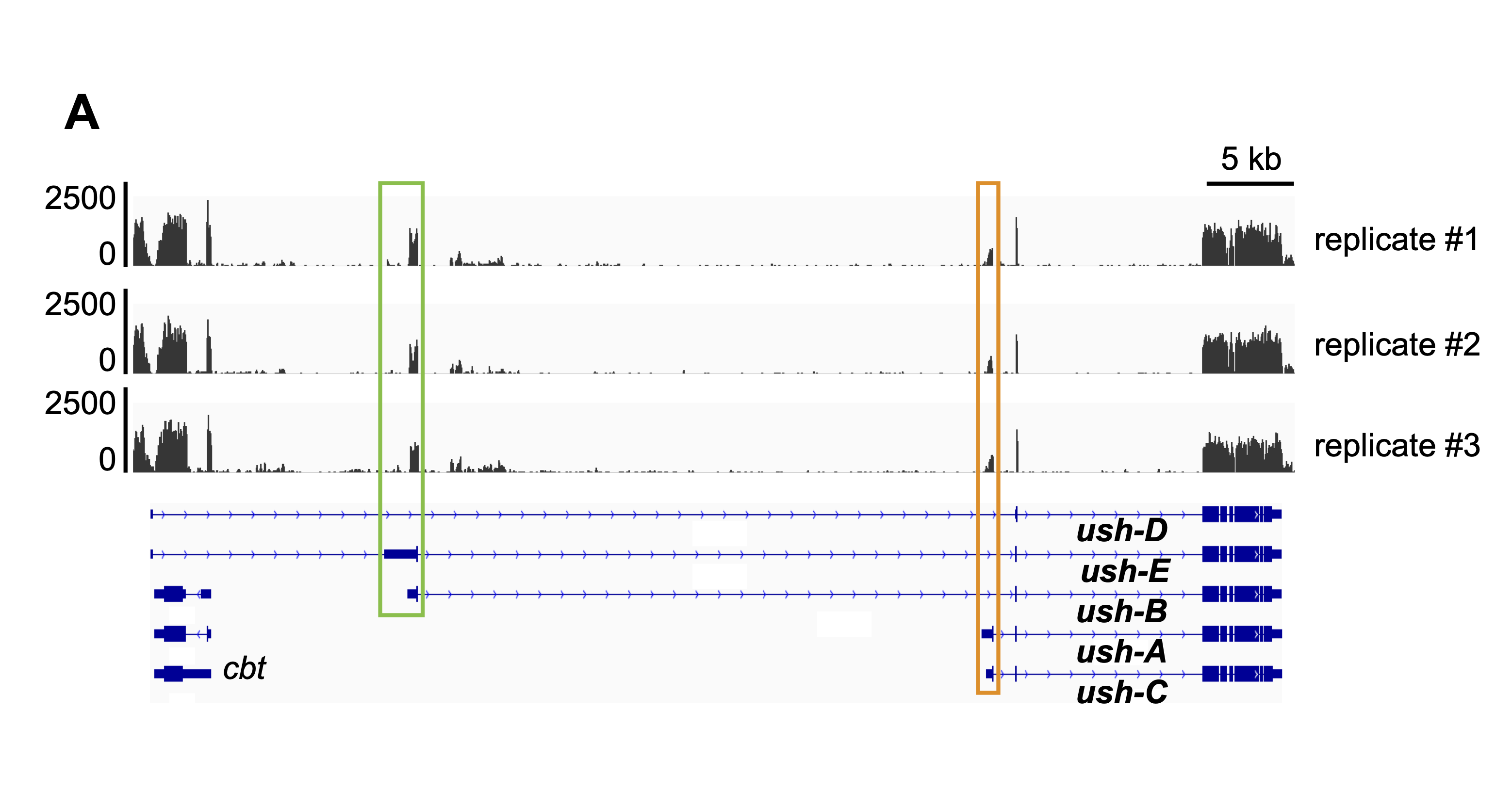

Supplement: S3 Fig — A Genome browser snapshots of the Ush gene locus displaying RNA-seq coverage in S2 cells from biological triplicates. Exons encoding unique N-termini are highlighted in green (Ush-B specific) and orange (Ush-A specific). (TIF) [file pgen.1009318.s003.tif]

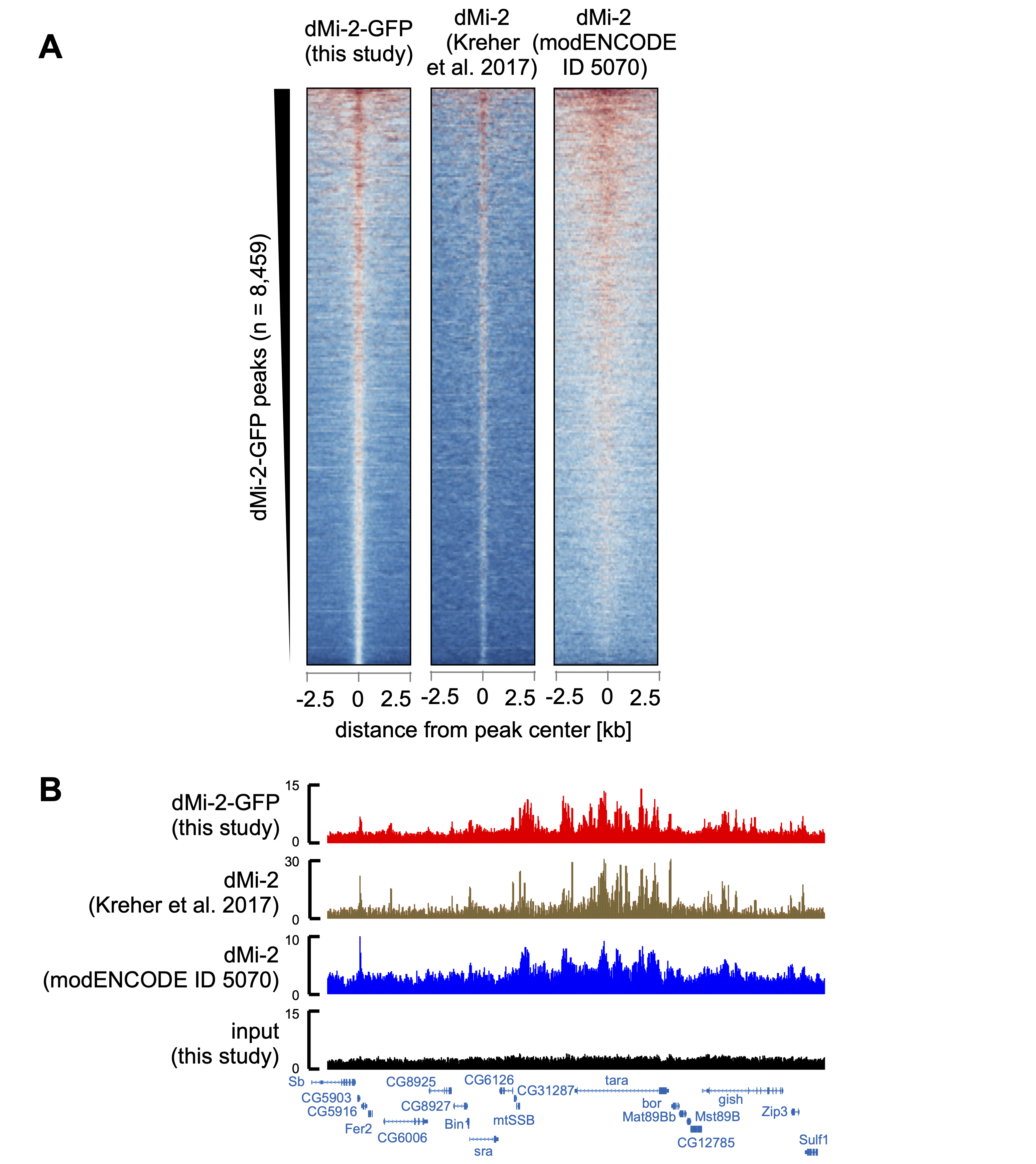

Supplement: S4 Fig — A dMi-2 ChIP-seq peaks obtained in this study were ranked and signals were compared to two other datasets (Kreher et al., 2017 and modENCODE ID 5070) in a region of 5 kb surrounding the respective peak. B Genome browser snapshots of an exemplary region displaying dMi-2 occupancy (red: this study; ochre: Kreher et al., 2017; blue: modENCODE ID 5070). Input signals of this study are shown in black. Location of genes is displayed below with boxes indicating exons. (TIF) [file pgen.1009318.s004.tif]

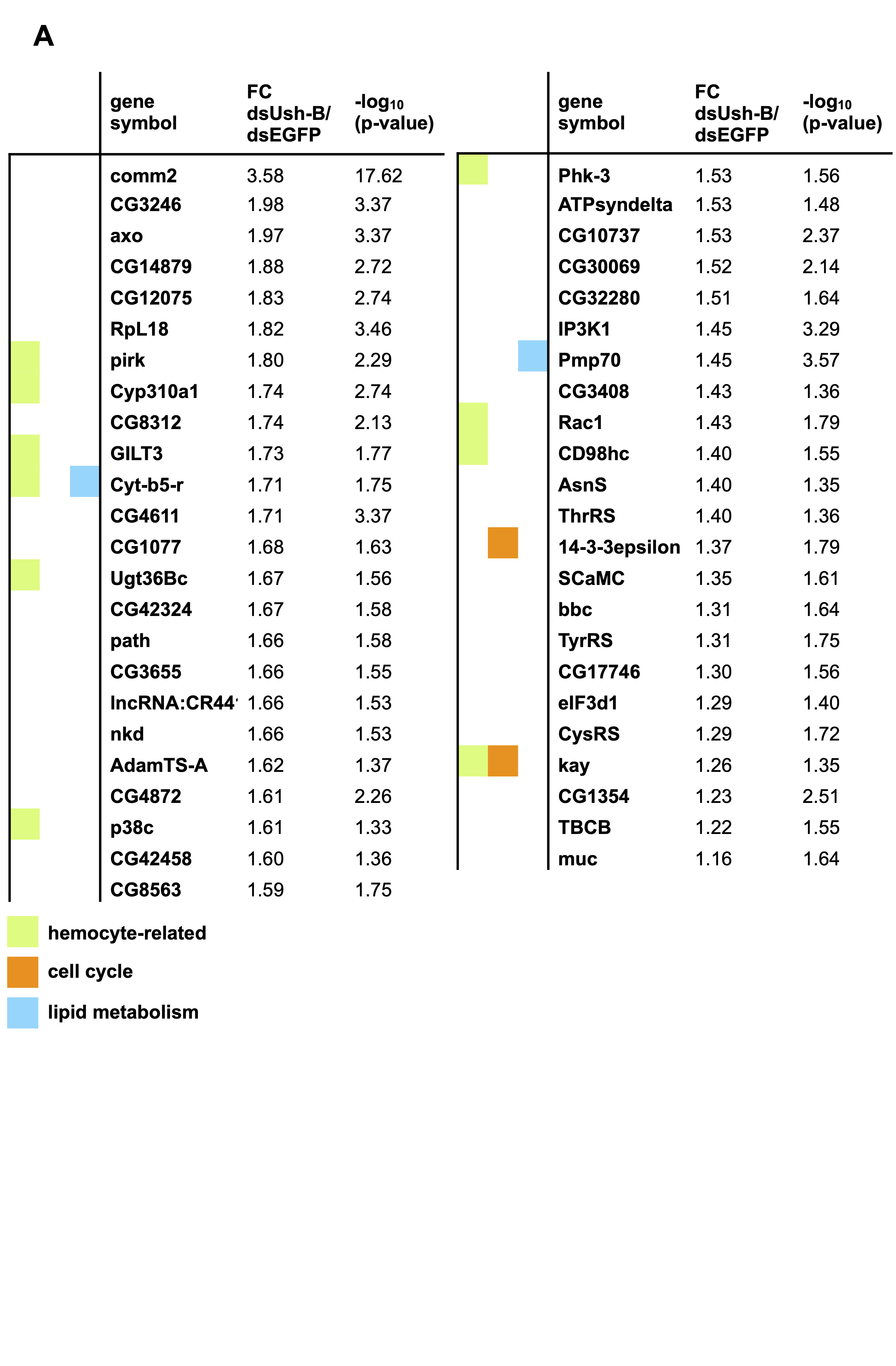

Supplement: S5 Fig — Tables of genes that are significantly upregulated (adj. p < 0.05) upon depletion of of Ush-B. Gene symbols are indicated along with the respective fold change relative to cells transfected with control dsRNA (dsEGFP). Respective -log10(p-values) are indicated in the last row. Coloured boxes mark genes associated with hemocyte functions or are specifically expressed in Drosophila hemocytes (green), genes associated with cell cycle (orange), and genes involved in lipid metabolism (blue). (TIF) [file pgen.1009318.s005.tif]

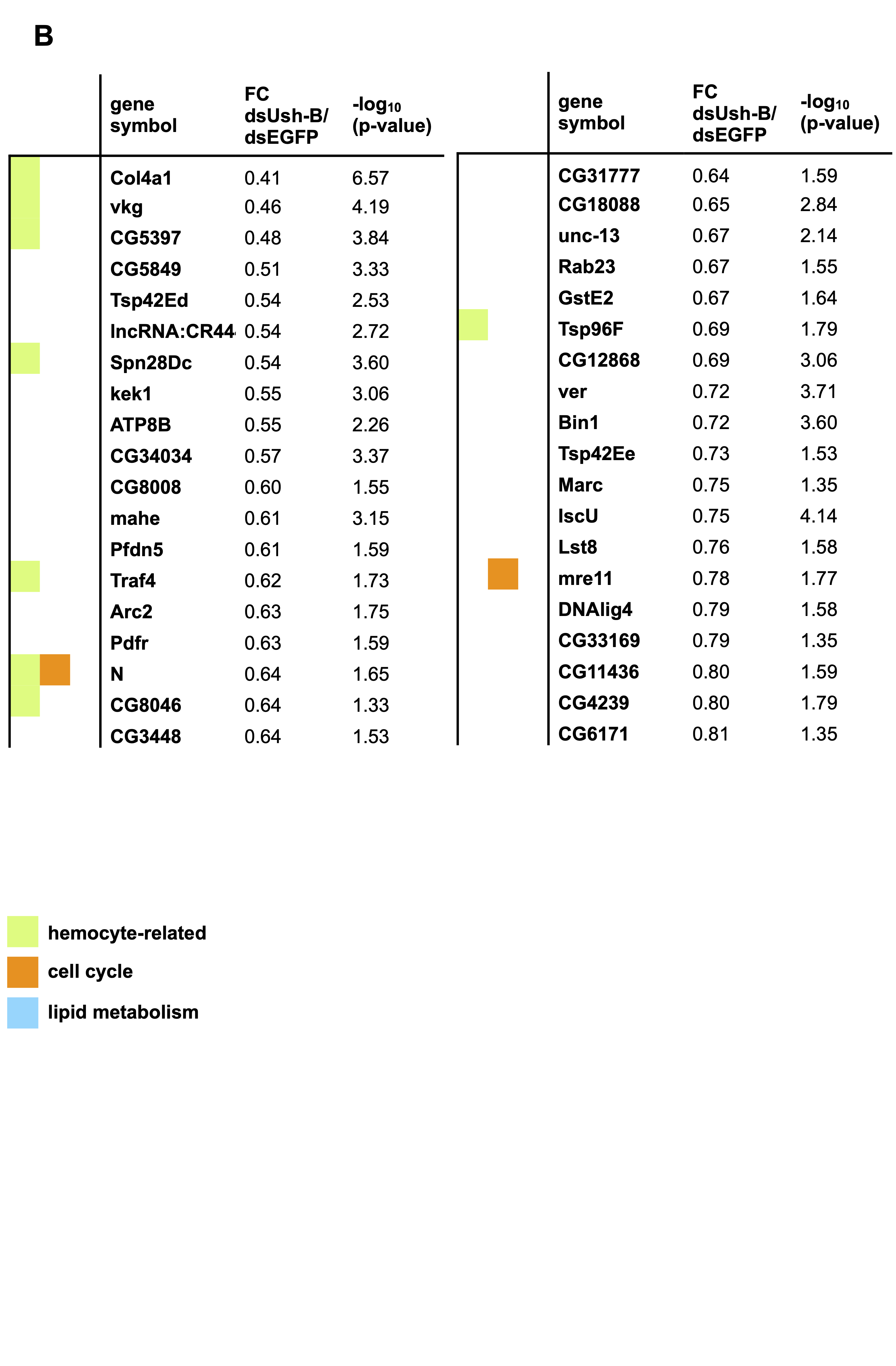

Supplement: S6 Fig — Tables of genes that are significantly downregulated (adj. p < 0.05) upon depletion of of Ush-B. Gene symbols are indicated along with the respective fold change relative to cells transfected with control dsRNA (dsEGFP). Respective -log10(p-values) are indicated in the last row. Coloured boxes mark genes associated with hemocyte functions or are specifically expressed in Drosophila hemocytes (green), genes associated with cell cycle (orange), and genes involved in lipid metabolism (blue). (TIF) [file pgen.1009318.s006.tif]

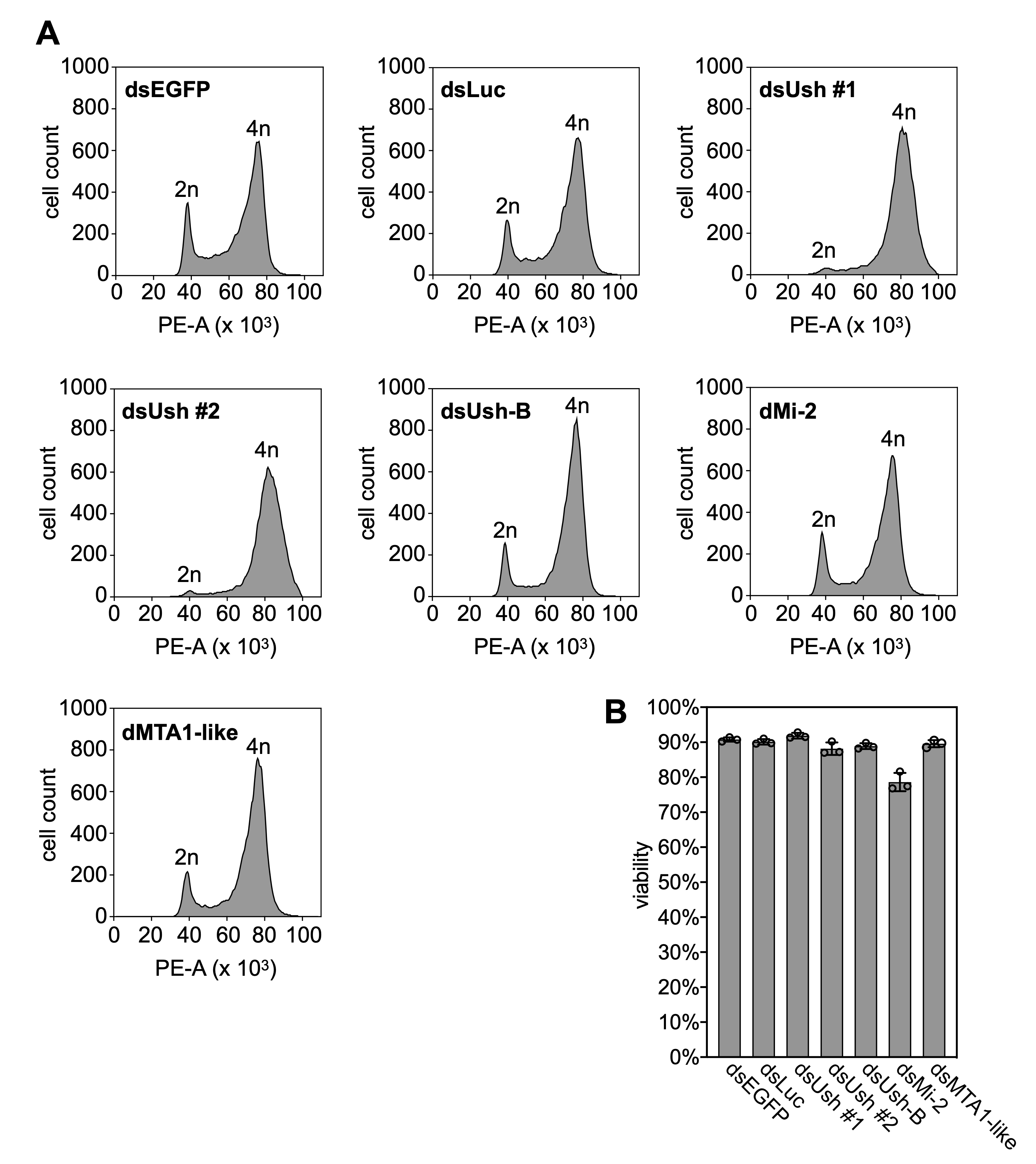

Supplement: S7 Fig — A Flow cytometry following PI-staining of S2 cells upon dsRNA-mediated depletion of indicated proteins. dsRNA-transfected cells were fixed, stained with PI and subjected to flow cytometry. Histograms show the number of cells plotted against the PI signal (Area of PE channel). The diploid cell population (2n) and cells that have undergone replication (4n) are indicated. Transfection of dsEGFP and dsLuc severd as control. Two different dsRNA constructs against Ush (all isoforms) were used (dsUsh #1 & dsUsh #2). B Viability assay of S2 cells upon depletion of indicated proteins. Viability of cells transfected with control dsRNA (dsEGFP and dsLuc) or dsRNA constructs targeting Ush (dsUsh #1 and dsUsh #2), Ush-B, dMi-2 and dMTA1-like was measured 96 hours post transfection. Error bars represent the standard deviation from biological triplicates (n = 3) and individual values are indicated with circles. (TIF) [file pgen.1009318.s007.tif]

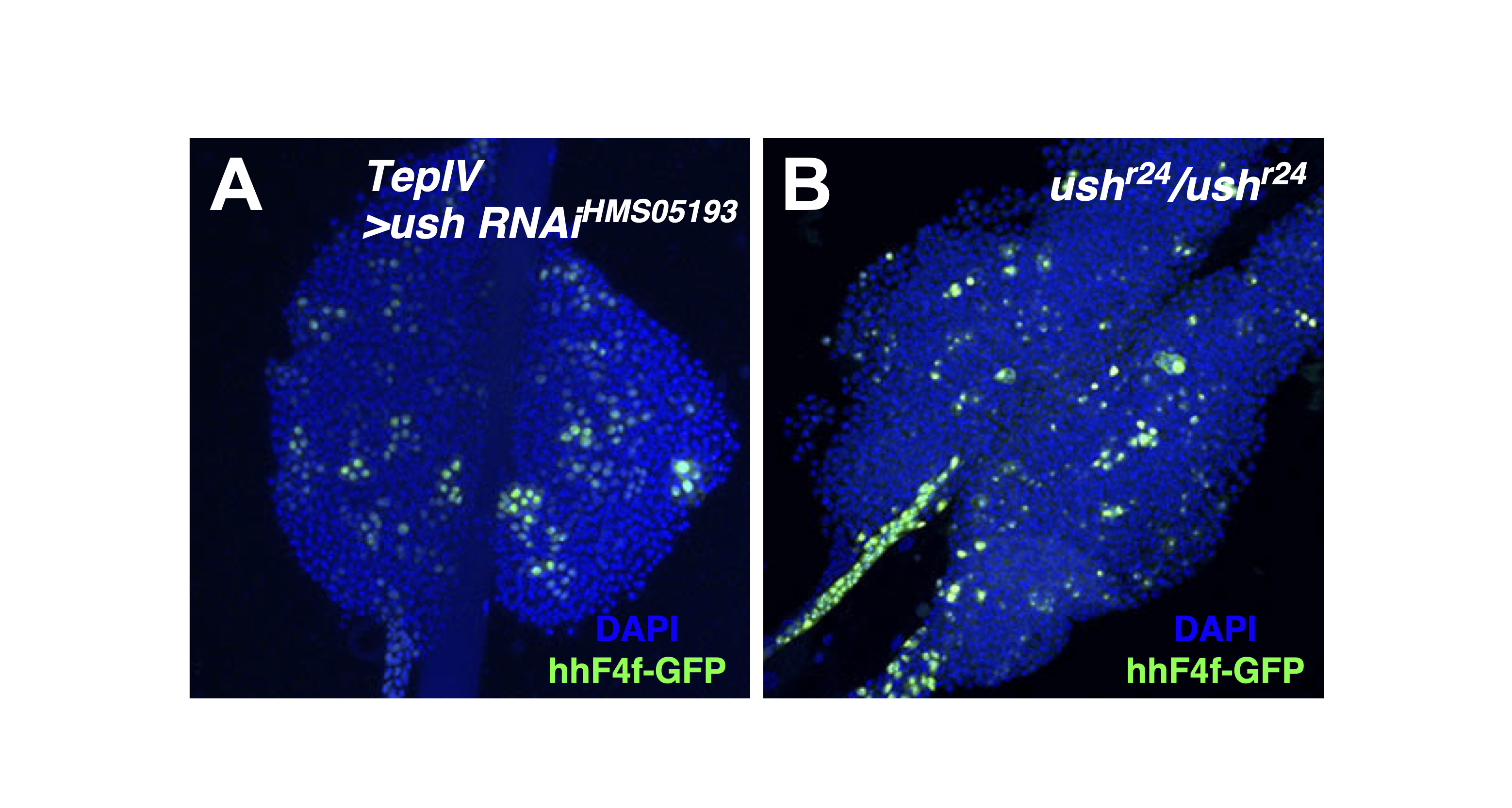

Supplement: S8 Fig — Lymph glands isolated from larvae that express a dsRNA against Ush in the medullary zone (A), or from larvae that carry homozygous Ush mutant alleles (B). All larvae carry a construct, reporting the activity of a minimal Hedgehog enhancer by GFP expression (hhF4f-GFP; green). (TIF) [file pgen.1009318.s008.tif]
