# Supplementary material for: Ush regulates hemocyte-specific gene expression, fatty acid metabolism and cell cycle progression and cooperates with dNuRD to orchestrate hematopoiesis
Source: PLoS Genet. 2021 Feb 18;17(2):e1009318. doi: 10.1371/journal.pgen.1009318 (PMC7891773; doi:10.1371/journal.pgen.1009318)
Supplement: S1 Table — Representative Ush-regulated genes of each gene class (investigated in Figs 2E and 6C) are listed. Columns 3 and 4 indicate binding of Ush and dMi-2 to the respective gene loci detected by anti-GFP ChIP sequencing (see Figs 1 and 5). (PDF) [file pgen.1009318.s009.pdf]

		annotated peak within promoter and/or gene body	
**Genes validated by RTqPCR**	**category**	**Ush-GFP**	**dMi-2-GFP**
**CG16267**	**hemocyte-related**	yes	yes
**pirk**	**hemocyte-related**	yes	yes
**GILT3**	**hemocyte-related**	yes	no
**Lozenge**	**hemocyte-related**	yes	no
**atilla**	**hemocyte-related**	no	no
**AurB**	**cell cycle**	no	no
**CHES-1**	**cell cycle**	yes	yes
**CDK1**	**cell cycle**	no	yes
**polo**	**cell cycle**	no	yes
**Cyclin B**	**cell cycle**	yes	yes
**Mcad**	**lipid metabolism**	yes	yes
**Echs1**	**lipid metabolism**	yes	yes
**ACC**	**lipid metabolism**	yes	yes
**CROT**	**lipid metabolism**	no	no
**fa2h**	**lipid metabolism**	yes	yes
