# Supplementary material for: Ush regulates hemocyte-specific gene expression, fatty acid metabolism and cell cycle progression and cooperates with dNuRD to orchestrate hematopoiesis
Source: PLoS Genet. 2021 Feb 18;17(2):e1009318. doi: 10.1371/journal.pgen.1009318 (PMC7891773; doi:10.1371/journal.pgen.1009318)
Supplement: S2 Table — List of genes that show significant changes (adj. p < 0.05) upon depletion of Ush-B. Gene identifiers, fold changes and p-values of each gene are listed. Genes were sorted into the groups “hemocyte-related” (green), “cell cycle” (orange) or “lipid metabolism” (blue) according to the references given in columns 10 and 11. (PDF) [file pgen.1009318.s010.pdf]

hemocyte-related	cell cycle	lipid metabolism	gene symbol	FlyBase ID	log_2(FC dsUsh-B/ dsEGFP)	FC dsUsh-B/ dsEGFP	$-log_{10}$(p-value)	p-value	FlyBase GO_Biological Function / additional references	expression pattern (BDGP)
X	X		**N**	FBgn0004647	-0.652	0.64	1.65	2.26E-02	regulation of crystal cell differentiation (GO:0042689); lamellocyte differentiation (GO:0035171); negative regulation of lamellocyte differentiation (GO:0035204); crystal cell differentiation (GO:0042688); larval lymph gland hemopoiesis (GO:0035167); embryonic crystal cell differentiation (GO:0035165); regulation of mitotic cell cycle (GO:0007346); positive regulation of G1/S transition of mitotic cell cycle (GO:1900087); hemocyte proliferation (GO:0035172); embryonic hemopoiesis (GO:0035162); positive regulation of cell population proliferation (GO:0008284); positive regulation of crystal cell differentiation (GO:0042691)	
X	X		**kay**	FBgn0001297	0.331	1.26	1.35	4.50E-02	positive regulation of biosynthetic processes of antibacterial peptides active against Gram-negative bacteria (GO:0006964); positive regulation of peptidoglycan recognition protein signaling pathway (GO:0061059); regulation of cyclin-dependent protein serine/threonine kinase activity involved in G2/M transition of mitotic cell cycle (GO:0031660)	plasmatocytes A (stage 11-12)
X		X	**Cyt-b5-r**	FBgn0000406	0.775	1.71	1.75	1.79E-02	lipid metabolic process (GO:0006629)	procrystal cells (stage 11-12), crystal cells (stage 13-16)
X			**Col4a1 / Cg25c**	FBgn0000299	-1.289	0.41	6.57	2.71E-07		plasmatocytes A (stage 11-12), plasmatocytes (stage 13-16)
X			**vkg**	FBgn0016075	-1.114	0.46	4.19	6.52E-05		plasmatocytes A (stage 11-12), plasmatocytes (stage 13-16)
X			**CG5397**	FBgn0031327	-1.068	0.48	3.84	1.44E-04		plasmatocytes (stage 13-16)
X			**Spn28Dc**	FBgn0031973	-0.876	0.54	3.60	2.48E-04	negative regulation of melanization defense response (GO:0035009)	
X			**Traf4**	FBgn0026319	-0.681	0.62	1.73	1.86E-02	defense response to Gram-negative bacterium (GO:0050829)	
X			**CG8046**	FBgn0033388	-0.646	0.64	1.33	4.64E-02	positive regulation of peptidoglycan recognition protein signaling pathway (GO:0061059); positive regulation of antimicrobial humoral response (GO:0002760); peptidoglycan transport (GO:0015835)	
X			**Tsp96F**	FBgn0027865	-0.541	0.69	1.79	1.64E-02		procrystal cells (stage 11-12)
X			**CD98hc**	FBgn0037533	0.489	1.40	1.55	2.83E-02		procrystal cells, plasmatocytes A (stage 11-12)
X			**Rac1**	FBgn0010333	0.520	1.43	1.79	1.64E-02	hemocyte development (GO:0007516); immune response-regulating cell surface receptor signaling pathway involved in phagocytosis (GO:0002433); hemocyte migration (GO:0035099); melanotic encypsulation of foreign target (GO:0035011)	
X			**Phk-3**	FBgn0035089	0.616	1.53	1.56	2.74E-02	response to bacterium (GO:0009617)	
X			**p38c**	FBgn0267339	0.690	1.61	1.33	4.64E-02	response to bacterium (GO:0009617)	
X			**Ugt36Bc**	FBgn0040260	0.742	1.67	1.56	2.74E-02		crystal cell SA (stage 9-10), procrystal cells (stage 11-12), crystal cell (stage 13-16)
X			**GILT3**	FBgn0039098	0.790	1.73	1.77	1.70E-02	Involved in the immune response to bacterial infection (Kongton et al., 2014)	
X			**Cyp310a1**	FBgn0032693	0.801	1.74	2.74	1.83E-03		plasmatocytes A (stage 11-12), plasmatocytes (stage 13-16)
X			**pirk**	FBgn0034647	0.847	1.80	2.29	5.07E-03	negative regulation of peptidoglycan recognition protein signaling pathway (GO:0061060)	
	X		**mre11**	FBgn0020270	-0.365	0.78	1.77	1.70E-02	mitotic G2 DNA damage checkpoint (GO:0007059); intra-S DNA damage checkpoint (GO:0031573)	
	X		**14-3-3epsilon**	FBgn0020238	0.451	1.37	1.79	1.62E-02	mitotic cell cycle checkpoint (GO:0007093); regulation of mitotic nuclear division (GO:0007088)	
		X	**Pmp70**	FBgn0031069	0.539	1.45	3.57	2.72E-04	long-chain fatty acid import into peroxisome (GO:0015910)	
			**CG5849**	FBgn0038897	-0.975	0.51	3.33	4.65E-04		
			**Tsp42Ed**	FBgn0029507	-0.889	0.54	2.53	2.97E-03		
			**lncRNA:CR44458**	FBgn0265651	-0.879	0.54	2.72	1.90E-03		
			**kek1**	FBgn0015399	-0.855	0.55	3.06	8.76E-04		
			**ATP8B**	FBgn0037989	-0.854	0.55	2.26	5.55E-03		
			**CG34034**	FBgn0054034	-0.822	0.57	3.37	4.22E-04		
			**CG8008**	FBgn0033387	-0.729	0.60	1.55	2.83E-02		

hemocyte-related	cell cycle	lipid metabolism	gene symbol	FlyBase ID	log_2(FC dsUsh-B/ dsEGFP)	FC dsUsh-B/ dsEGFP	$-log_{10}$(p-value)	p-value	FlyBase GO_Biological Function / additional references	expression pattern (BDGP)
			**mahe**	FBgn0029979	-0.722	0.61	3.15	7.07E-04		
			**Pfdn5 / CG7048**	FBgn0038976	-0.714	0.61	1.59	2.58E-02		
			**Arc2**	FBgn0033928	-0.668	0.63	1.75	1.79E-02		
			**Pdfr**	FBgn0260753	-0.665	0.63	1.59	2.58E-02		
			**CG3448**	FBgn0035996	-0.640	0.64	1.53	2.94E-02		
			**CG31777**	FBgn0051777	-0.634	0.64	1.59	2.55E-02		
			**CG18088**	FBgn0032082	-0.627	0.65	2.84	1.44E-03		
			**unc-13**	FBgn0025726	-0.582	0.67	2.14	7.22E-03		
			**Rab23**	FBgn0037364	-0.574	0.67	1.55	2.84E-02		
			**GstE2**	FBgn0063498	-0.568	0.67	1.64	2.30E-02		
			**CG12868**	FBgn0033945	-0.534	0.69	3.06	8.76E-04		
			**ver**	FBgn0262524	-0.471	0.72	3.71	1.94E-04		
			**Bin1**	FBgn0024491	-0.469	0.72	3.60	2.48E-04		
			**Tsp42Ee**	FBgn0029506	-0.445	0.73	1.53	2.95E-02		
			**Marc / CG1665**	FBgn0033451	-0.415	0.75	1.35	4.46E-02		
			**IscU**	FBgn0037637	-0.407	0.75	4.14	7.28E-05		
			**Lst8**	FBgn0264691	-0.397	0.76	1.58	2.64E-02		
			**DNAlig4 / Lig4**	FBgn0030506	-0.344	0.79	1.58	2.64E-02		
			**CG33169**	FBgn0053169	-0.342	0.79	1.35	4.46E-02		
			**CG11436**	FBgn0029713	-0.319	0.80	1.59	2.55E-02		
			**CG4239**	FBgn0030745	-0.313	0.80	1.79	1.64E-02		
			**CG6171**	FBgn0026737	-0.300	0.81	1.35	4.47E-02		
			**muc**	FBgn0283658	0.218	1.16	1.64	2.30E-02		
			**TBCB**	FBgn0034451	0.282	1.22	1.55	2.83E-02		
			**CG1354**	FBgn0030151	0.293	1.23	2.51	3.09E-03		
			**CysRS / Aats-cys**	FBgn0027091	0.363	1.29	1.72	1.92E-02		
			**eIF3d1 / eIF-3p66**	FBgn0040227	0.371	1.29	1.40	4.02E-02		
			**CG17746**	FBgn0035425	0.376	1.30	1.56	2.74E-02		
			**TyrRS /Aats-tyr**	FBgn0027080	0.389	1.31	1.75	1.77E-02		
			**bbc**	FBgn0033844	0.390	1.31	1.64	2.30E-02		
			**SCaMC**	FBgn0052103	0.431	1.35	1.61	2.46E-02		
			**ThrRS / Aats-thr**	FBgn0027081	0.483	1.40	1.36	4.37E-02		
			**AsnS**	FBgn0270926	0.484	1.40	1.35	4.48E-02		
			**CG3408**	FBgn0036008	0.520	1.43	1.36	4.34E-02		
			**IP3K1**	FBgn0032147	0.541	1.45	3.29	5.11E-04		
			**CG32280**	FBgn0052280	0.595	1.51	1.64	2.30E-02		
			**CG30069**	FBgn0050069	0.605	1.52	2.14	7.22E-03		
			**CG10737**	FBgn0034420	0.612	1.53	2.37	4.23E-03		
			**ATPsyndelta**	FBgn0028342	0.614	1.53	1.48	3.35E-02		

hemocyte-related	cell cycle	lipid metabolism	gene symbol	FlyBase ID	log_2(FC dsUsh-B/ dsEGFP)	FC dsUsh-B/ dsEGFP	$-log_{10}$(p-value)	p-value	FlyBase GO_Biological Function / additional references	expression pattern (BDGP)
			**CG8563**	FBgn0035777	0.665	1.59	1.75	1.77E-02		
			**CG42458**	FBgn0259935	0.675	1.60	1.36	4.34E-02		
			**CG4872**	FBgn0030799	0.691	1.61	2.26	5.55E-03		
			**AdamTS-A**	FBgn0286071	0.700	1.62	1.37	4.29E-02		
			**nkd**	FBgn0002945	0.727	1.66	1.53	2.96E-02		
			**lncRNA:CR44138**	FBgn0264987	0.728	1.66	1.53	2.94E-02		
			**CG3655**	FBgn0040397	0.729	1.66	1.55	2.83E-02		
			**path**	FBgn0036007	0.731	1.66	1.58	2.62E-02		
			**CG42324**	FBgn0259224	0.738	1.67	1.58	2.64E-02		
			**CG1077**	FBgn0037405	0.748	1.68	1.63	2.37E-02		
			**CG4611**	FBgn0035591	0.774	1.71	3.37	4.22E-04		
			**CG8312**	FBgn0037720	0.796	1.74	2.13	7.33E-03		
			**RpL18**	FBgn0035753	0.860	1.82	3.46	3.44E-04		
			**CG12075**	FBgn0030065	0.869	1.83	2.74	1.84E-03		
			**CG14879**	FBgn0038419	0.908	1.88	2.72	1.92E-03		
			**axo**	FBgn0262870	0.978	1.97	3.37	4.22E-04		
			**CG3246**	FBgn0031538	0.983	1.98	3.37	4.22E-04		
			**comm2**	FBgn0041160	1.841	3.58	17.62	2.42E-18		
