# Supplementary material for: Ush regulates hemocyte-specific gene expression, fatty acid metabolism and cell cycle progression and cooperates with dNuRD to orchestrate hematopoiesis
Source: PLoS Genet. 2021 Feb 18;17(2):e1009318. doi: 10.1371/journal.pgen.1009318 (PMC7891773; doi:10.1371/journal.pgen.1009318)
Supplement: S3 Table — Total numbers of examined larvae and penetrance levels of increased lamellocyte counts associated with Fig 7H. Genotypes and the affected dNuRD complex subunit are listed in columns 1-2. (PDF) [file pgen.1009318.s011.pdf]

NuRD complex subunit	Genotype crossed to y w;ush^{VX22}, MSN-C/CyO y+ or y w; MSN-C	genetic background (selected based on MSN-C, y and/or Tb phenotypic markers)	number of MSN larvae tested	number of MSN larvae with ≥10x lm*	% larvae w/ ≥10x lm*	Estimated penetrance
	w^{1118}	y w;ush^{VX22}, MSN-C/+	32	3	9.4	9.4
	y; ush^{r24}/CyO y+ #	y w;ush^{VX22}, MSN-C/ush^{r24}	14	14	100.0	100.0
dMTA-1like	w^{1118}; MTA1-like^{d09140}/TM6B, Tb^{1} +	w^{1118}/yw; MSN-C/+; MTA1-like^{d09140}/+	28	2	7.1	7.1
		w^{1118}/yw; ush^{VX22}, MSN-C/+; MTA1-like^{d09140}/+	48	25	52.1	52.1
	y^{1} w*; MTA1-like^{MI01790} **	y w; MSN-C/+; MTA1-like^{MI01790}/+	31	1	3.2	3.2
		y w;ush^{VX22}, MSN-C/+; MTA1-like^{MI01790}/+	38	28	73.7	73.7
dp66	w^{1118}; simj^{BG00403}/TM6B, Tb^{1} +	w^{1118}/yw; MSN-C/+; simj^{BG00403}/+	26	0	0.0	0.0
		w^{1118}/y w;ush^{VX22}, MSN-C/+; simj^{BG00403}/+	39	17	43.6	43.6
	simj^{01814} ry^{506} **	y w/+; MSN-C/+; simj^{01814} ry^{506}/+	40	2	5.0	5.0
		y w/+;ush^{VX22}, MSN-C/+; simj^{01814} ry^{506}/+	61	31	50.8	50.8
dRpd3	y^{1} w*; HDAC1$^{12-37}$/TM6B, Tb^{1} *	y w; MSN-C/+; HDAC1$^{12-37}$ /+	30	15	50.0	50.0
		y w;ush^{VX22}, MSN-C/+; HDAC1$^{12-37}$ /+	42	35	83.3	83.3
	HDAC1^{04556} ry^{506}/TM3, ry^{RK} Sb^{1} Ser^{1} ++	y w/+; MSN-C/+; HDAC1^{04556} ry^{506} /+ AND y w/+; MSN-C/+; TM3, ry^{RK} Sb^{1} Ser^{1} /+	27	2	7.4	14.8
		y w/+;ush^{VX22}, MSN-C/+; HDAC1^{04556} ry^{506} /+ AND y w/+;ush^{VX22}, MSN-C/+; TM3, ry^{RK} Sb^{1} Ser^{1} /+	53	17	32.1	64.2
dMi-2	Mi-2^{4} red^{1} e^{4}/TM6B, Sb^{1} Tb^{1} ca^{1} +	y w/+; MSN-C/+; Mi-2^{4} red^{1} e^{4}/+	NT	NT	NT	NT
		y w/+;ush^{VX22}, MSN-C/+; Mi-2^{4} red^{1} e^{4}/+	45	16	35.6	35.6
	y^{1} w^{1118}; Mi-2^{L1243}/TM3, Ser^{1} ++	y w; MSN-C/+; Mi-2^{L1243}/+ AND y w; MSN-C/+; TM3, Ser^{1}/+	NT	NT	NT	NT
		y w;ush^{VX22}, MSN-C/+; Mi-2^{L1243}/+ AND y w;ush^{VX22}, MSN-C/+; TM3, Ser^{1}/+	51	8	15.7	31.4

*Estimated number of lamellocytes (lm)

#To identify animals with the ush^{r24}/ ush^{vx22}, MSN genotype, larvae with yellow mouth hooks were selected.

+The Tb balancer chromosome is easily distinguished from the MTA-1 like chromosome.

**Homozygous viable

++Heterozygous, which assumes only 50% of the animals that carry the *ush; MSN-mCherry* chromosome are double heterozygotes and thus, the estimated penetrance may be twice the value of the % larvae with increased lamellocyte differentiation.

NT: not tested
