# Supplementary material for: Ush regulates hemocyte-specific gene expression, fatty acid metabolism and cell cycle progression and cooperates with dNuRD to orchestrate hematopoiesis
Source: PLoS Genet. 2021 Feb 18;17(2):e1009318. doi: 10.1371/journal.pgen.1009318 (PMC7891773; doi:10.1371/journal.pgen.1009318)
Supplement: S4 Table — List of all oligonucleotides and primers used in this study. Sequences and applications are given. References are indicated in column 5. (PDF) [file pgen.1009318.s012.pdf]

name	sequence	application	method
**sgRNA_scaffold**	GTTTAAGAGCTATGCTGGAAACAGCATAGCAAGTTTAAATAAGGCTAGTCCGTTATCA ACTTGAAAAAGTGGCACCGAGTCGGTGC	general sgRNA template synthesis	endogenous tagging
**U6-promotor_s**	GCTCACCTGTGATTGCTCCTAC	general sgRNA template synthesis	endogenous tagging
**sgRNA_as**	gcttattctcAAAAAAGCACCGACTCGGTGCCACT	general sgRNA template synthesis	endogenous tagging
**lig4_RNAi_s**	taatacgactcactatagggCCCAATGATCCAAAGTGTTTTTGCA	generation of dsRNA against lig4	endogenous tagging
**lig4_RNAi_as**	taatacgactcactatagGGAAGTAGGATGCCTTCGCGA	generation of dsRNA against lig4	endogenous tagging
**mus308_RNAi_s**	taatacgactcactataggGCTGGGACTCCACCGGAAAG	generation of dsRNA against mus308	endogenous tagging
**mus308_RNAi_as**	taatacgactcactatagggTACCGTCGCCGTCCAGTAATG	generation of dsRNA against mus308	endogenous tagging
**CRISPR_Mi2_Ct**	cctattttcaatttaacgtcgTCGAATAATTCCGGCGTCTgtttaagagctatgctg	sgRNA synthesis dMi-2 C-term	endogenous tagging of dMi-2
**Mi2_Ctag_s**	TTTGCCAACTTTCGGCCACAGTTCTCGGTGCCCGGCCAGCTATCGAATAATTCCGGCG TCggatcttccggatggctcgag	generation of homology donors for dMi-2	endogenous tagging of dMi-2
**Mi2_Ctag_as**	TTGAGTAAAGTATATTTGCATGGAATACGAATGCTCTTAACATAGATATTGAGGAGAT GCgaagttcctattctctagaaagtataggaacttccatatg	generation of homology donors for dMi-2	endogenous tagging of dMi-2
**Mi2_PCR_C_us_s**	tgccagcattcacgagcggaccg	genotyping of cell lines containing tagged dMi-2 alleles	endogenous tagging of dMi-2
**Mi2_PCR_C_ds_as**	gtgggtgtgcaggtgtgttaccgtg	genotyping of cell lines containing tagged dMi-2 alleles	endogenous tagging of dMi-2
**CRISPR_ush_Ct**	cctattttcaatttaacgtcgCATTTGAGAAAGCCAGCTGgtttaagagctatgctg	sgRNA synthesis Ush C-term	endogenous tagging of Ush
**ush_Ctag_s**	GGCCTGGTCGGCGGACACGGCCAGCAGAAGAACAAGGAAAACCTGCAGGAGGCGGCCA TTggatcttccggatggctcgag	generation of homology donors for Ush	endogenous tagging of Ush
**ush_Ctag_as**	GAAGCACGTGTAATACCACTCAAGCTGCTTGCGCTGCGCCTCCCACCGCAGCTGGCTT TCgaagttcctattctctagaaagtataggaacttccatatg	generation of homology donors for Ush	endogenous tagging of Ush
**ush_PCR_us_s**	AGTCCGAATCACCTGGGCGGAGG	genotyping of cell lines containing tagged Ush alleles	endogenous tagging of Ush
**ush_PCR_ds_as**	GAGGCTAGGATTCGATTAGTTTCGA	genotyping of cell lines containing tagged Ush alleles	endogenous tagging of Ush
**EGFP-T7-RNAi-fw**	gaattaatacgactcactatagggaGAGCTGGACGGCGACGTAA	generation of dsRNA against EGFP	RNAi
**EGFP-T7-RNAi-rv**	gaattaatacgactcactatagggagACTTGTACAGCTCGTCCATG	generation of dsRNA against EGFP	RNAi
**Luc-T7-RNAi-fw**	taatacgactcactatagggCTGGTTCCTGGAACAATTGC	generation of dsRNA against Luciferase	RNAi
**Luc-T7-RNAi-rv**	taatacgactcactatagggTGACGAACGTGTACATCGA	generation of dsRNA against Luciferase	RNAi
**Ush-T7-RNAi-1-fw**	taatacgactcactatagggCAGCCCAAGCACTCCG	generation of dsRNA against Ush (#1)	RNAi
**Ush-T7-RNAi-1-rv**	taatacgactcactatagggGCTGTAGGAGCACTGG	generation of dsRNA against Ush (#1)	RNAi
**Ush-T7-RNAi-2-fw**	taatacgactcactatagggACACTTCCCTGGACAACCTG	generation of dsRNA against Ush (#2)	RNAi
**Ush-T7-RNAi-2-rv**	taatacgactcactatagggAGTTGTGGTAGATGCCCCTG	generation of dsRNA against Ush (#2)	RNAi
**Mi-2-T7-RNAi-fw**	taatacgactcactatagggTTAACTCGCTGACCAAGGCT	generation of dsRNA against dMi-2	RNAi
**Mi-2-T7-RNAi-rv**	taatacgactcactatagggATATCGTTGTGGGGATTCCA	generation of dsRNA against dMi-2	RNAi
**Ush-B-T7-RNAi-fw**	taatacgactcactatagggGCTTCGAACGGACGTCTTTA	generation of dsRNA against Ush-B	RNAi
**Ush-B-T7-RNAi-rv**	taatacgactcactatagggGGGCAATCAATGCGATTACT	generation of dsRNA against Ush-B	RNAi
**MTA-T7-RNAi-fw**	taatacgactcactatagggCAGAACGCGAGACAACAAAA	generation of dsRNA against dMTA1-like	RNAi
**MTA-T7-RNAi-rv**	taatacgactcactatagggTGGAACTTTAGAGCGCGATT	generation of dsRNA against dMTA1-like	RNAi

name	sequence	application	method
**rp49-RT-fw**	TGTCCTTCCAGCTTCAAGATGACCATC	amplification of rp49 cDNA fragment in qPCR	RT-qPCR
**rp49-RT-rv**	CTTGGGCTTGCGCCATTTGTG	amplification of rp49 cDNA fragment in qPCR	RT-qPCR
**CG16762-RT-fw**	GGTATCGATGCCGACTTCC	amplification of CG16762 cDNA fragment in qPCR	RT-qPCR
**CG16762-RT-rv**	TGGAGCCCACATTGGCA	amplification of CG16762 cDNA fragment in qPCR	RT-qPCR
**lz-RT-fw**	CGAATTGGTGCGCACGAG	amplification of Lozenge cDNA fragment in qPCR	RT-qPCR
**lz-RT-rv**	CCCGGATGGTGACATAGGTG	amplification of Lozenge cDNA fragment in qPCR	RT-qPCR
**pirk-RT-fw**	AGCGGCGATGCCAAGAAAAG	amplification of pirk cDNA fragment in qPCR	RT-qPCR
**pirk-RT-rv**	GCTCCGTGCCGTATCGTTAG	amplification of pirk cDNA fragment in qPCR	RT-qPCR
**GILT3-RT-fw**	CAAGGCGGGGTTCTACAACA	amplification of GILT3 cDNA fragment in qPCR	RT-qPCR
**GILT3-RT-rv**	TCCGAATGTCCAGGGTCTCAA	amplification of GILT3 cDNA fragment in qPCR	RT-qPCR
**atilla-RT-fw**	AAACAAGTGATTTTCGTGCTCCT	amplification of atilla cDNA fragment in qPCR	RT-qPCR
**atilla-RT-rv**	CGCGGATGTTAGAGGCAGA	amplification of atilla cDNA fragment in qPCR	RT-qPCR
**AurB-RT-fw**	TACGGACAGCCATACGATTGGAG	amplification of Aurora B cDNA fragment in qPCR	RT-qPCR
**AurB-RT-rv**	ACCAGATAGTGCGAGTGGCG	amplification of Aurora B cDNA fragment in qPCR	RT-qPCR
**CHES-1-RT-fw**	CAGTGAGGAGAATCACAACATCAC	amplification of CHES-1 cDNA fragment in qPCR	RT-qPCR
**CHES-1-RT-rv**	CTGAGCCGCACTCCACAATC	amplification of CHES-1 cDNA fragment in qPCR	RT-qPCR
**Cdk1-RT-fw**	CCGCGATCAGAGAAATTTCGTTG	amplification of Cdk1 cDNA fragment in qPCR	RT-qPCR
**Cdk1-RT-rv**	GAGGTCCATCGATAGGAATTCAAAG	amplification of Cdk1 cDNA fragment in qPCR	RT-qPCR
**polo-RT-fw**	TCTGCACGACGCCATTACCG	amplification of polo cDNA fragment in qPCR	RT-qPCR
**polo-RT-rv**	AGAATTCGCGGCTTTCCGTT	amplification of polo cDNA fragment in qPCR	RT-qPCR
**CycB-RT-fw**	CCCACTAAAGTTACAGTCAAGTCC	amplification of Cyclin B cDNA fragment in qPCR	RT-qPCR
**CycB-RT-rv**	CTGAAACTCCCATCACGGGT	amplification of Cyclin B cDNA fragment in qPCR	RT-qPCR
**Mcad-RT-fw**	GTTTGGATCATTTCGCTGGCA	amplification of Mcad cDNA fragment in qPCR	RT-qPCR
**Mcad-RT-rv**	CAGCAAGCTTGTTGAGGAAC	amplification of Mcad cDNA fragment in qPCR	RT-qPCR
**Echs1-RT-fw**	ATCGGCACCCACTCCAATCT	amplification of Echs1 cDNA fragment in qPCR	RT-qPCR
**Echs1-RT-rv**	CTTGCGATCGGCCGTGGAG	amplification of Echs1 cDNA fragment in qPCR	RT-qPCR
**ACC-RT-fw**	ACAAGATTGGCTTCCCCGTAATG	amplification of ACC cDNA fragment in qPCR	RT-qPCR
**ACC-RT-rv**	GCTTGAACCTGGCGGAACAG	amplification of ACC cDNA fragment in qPCR	RT-qPCR
**CROT-RT-fw**	TGCATAAAGAAATTGCTCCCAC	amplification of CROT cDNA fragment in qPCR	RT-qPCR
**CROT-RT-rv**	TTGTTGGATGACGCCCTCAG	amplification of CROT cDNA fragment in qPCR	RT-qPCR
**fa2h-RT-fw**	GATAGTATGGAGCACCTAGTGGAC	amplification of fa2h cDNA fragment in qPCR	RT-qPCR
**fa2h-RT-rv**	CCAAGGGTCAAAGAGACGCA	amplification of fa2h cDNA fragment in qPCR	RT-qPCR
