# Supplementary material for: Ush regulates hemocyte-specific gene expression, fatty acid metabolism and cell cycle progression and cooperates with dNuRD to orchestrate hematopoiesis
Source: PLoS Genet. 2021 Feb 18;17(2):e1009318. doi: 10.1371/journal.pgen.1009318 (PMC7891773; doi:10.1371/journal.pgen.1009318)
Supplement: Attachment — Submitted filename: Response to reviewers - Lenz et al..docx [file pgen.1009318.s013.docx]

**Response to reviewers**

We thank all three reviewers for the time and effort they have put into evaluating our manuscript. We are grateful for the many comments and suggestion which we have all addressed. We feel that this has significantly improved our manuscript. In order to address the reviewers’ concerns we have performed a number of experiments and have generated numerous new figures which either have been incorporated into the revised version of our manuscript or are shown in this rebuttal letter. In general, we feel that figures shown in this rebuttal letter do not warrant inclusion in the manuscript which already contains more than 50 panels across main and supplementary figures. However, if the reviewers feel that additional figures should be included we would be happy to accommodate this wish.

**Reviewer #1**

We thank the reviewer for her/his comments. We appreciate the time and effort that went into many suggestions that aim to improve our manuscript.

*1. Given the unexpected results with the cell cycle genes, it would be very valuable to extend to in vivo and test whether the regulation seen in S2 cells is relevant in the pro-hemocytes, by testing some of the targets in a similar way to in Figure 7 and monitoring effects on proliferation.*

We agree that the regulation of Ush on cell cycle genes in S2 cells was unexpected. It is well established that Ush loss of function leads to tumour-like hyperproliferation in the lymph gland (Sorrentino *et al.*, 2007). Thus, Ush appears to affect cell division in opposite ways in (embryo-derived) S2 cells and larval lymph glands. In contrast to the S2 cell line which resembles a single, albeit poorly defined, pro-hemocyte cell type, the lymph gland is composed of multiple progenitor, differentiating and differentiated hemocytes (both plasmatocytes and crystal cells) in addition to the cells of the posterior signaling center. Differentiation and proliferation in the lymph gland is determined by both cell autonomous mechanisms as well as by extensive signalling between the different cell populations. Which cells hyperproliferate in the lymph glands of Ush mutants and if this is a direct (cell autonomous) or indirect (mediated by signaling) effect is not known. To decipher the effect of Ush on the cell cycle in the lymph gland would require a systematic modulation of Ush activity and the determination of changes in the expression of cell cycle genes in the many different cell types / differentiation stages the lymph gland contains. While this is doable in principle (scRNA-seq would be the most systematic and comprehensive approach) and would likely yield very interesting results, we strongly feel that this is beyond the scope of our present study.

*2. On what basis are the genes in Fig. 2E/6C categorized as hemocyte-related? Two appear to be implicated in immune response but it’s not obvious that others have hemocyte expression/function.*

These genes were categorized as "hemocyte-related" based on their expression and established roles in hemocyte function. This info is summarised in the table below. Please, note that we have removed Gs2 and Ugt36Bc (greyed out in table below) from Figures 2E and 6C and have included Lozenge and Attila instead.

| **hemocyte gene** | **name** | **reference** |
| --- | --- | --- |
| **CG16762** |  | target of IMD pathway (Lamianle et al., 2016, PNAS) |
| **Gs2** | Glutamine synthetase 2 | Gs1 (and Glutamate-dependend Redox system in general) involved in blood cell phagocytosis (Gonzalez et al., 2013, Curr Biol) |
| **pirk** | **p**oor **I**md **r**esponse upon **k**nock-in | negative regulation of peptidoglycan recognition protein signaling pathway (inferred from Flybase GO term: Biological function) |
| **GILT3** | **g**amma-interferon-**i**nducible **l**ysosomal **t**hiol reductase | involved in immune response to bacterial infection (Kongton et al., 2014, Dev Comp Immunol) |
| **Ugt36Bc** | UDP-glycosyltransferase family 37 member D1 | expressed in crystal cells and crystal cell progenitors (inferred from BDGP) |
| **Lozenge** |  | RUNX1 homolog, functions in crystal cell differentiation and wound healing (Fossett and Schulz, 2001, Differentiation) |
| **Attila** | L1 antigene | Marker for lamellocytes (Cattenoz et al., 2020, EMBO J) |

*It would be of major interest if some of the key differentiation genes were regulated by Ush-Mi2. For this reason, the data for Lozenge (a pro-crystal cell gene) in Supplementary Fig 2 should be included in the main figures. Is this also bound by Mi-2? Does Mi-2/MTA depletion cause derepression? That would add significant weight to the conclusions. Conversely, Msn expression changes in vivo are shown in Fig 7: is there evidence for Ush ± Mi-2 binding to Msn?*

We thank the reviewer for her/his suggestion and agree that inclusion of classical hemocyte differentiation genes would be of greater interest than some of the genes that we had chosen. We have now removed the *Gs2* and *Ugt36Bc* RTqPCR data from Figure 2E and replaced it with data for *Lozenge* (a crystal cell gene) and *Attila* (a lamellocyte gene). Both genes are upregulated upon depletion of Ush in S2 cells. Since the *Lozenge* RTqPCR data is now part of main Figure 2E, we have removed this data from Supplementary Figure 2. Supplementary Figure 2 now shows ChIPseq genome browser shots for Ush binding to the *Lozenge* and *Attila* loci. *Lozenge* but not *Attila* is bound by Ush. We have not detected binding of dMi-2 to either Lozenge or Attila in our ChIPseq analysis.

We have now also determined expression of both genes following depletion of dMi-2 and depletion of Ush-B. These data are now included in the revised Figure 6C. While we see significant upregulation of *Lozenge* following dMi-2 depletion this is not the case for *Attila*.

Finally, our ChIPseq analysis does indeed detect binding of dMi-2 (and Ush) to the *Msn* locus in S2 cells (see figure below).

It is important to point out that we do not claim that all Ush-regulated genes are (a) direct targets, (b) co-bound and (c) co-regulated by Ush and dMi-2. Nor would it be expected that S2 cells which were originally derived from embryonic pro-hemocytes and maintained in culture for decades would recapitulate precisely the regulation of hemocyte differentiation genes that can be observed in the complex lymph gland (see also our discussion of the differences between S2 cells and the lymph gland above). What we do claim is that Ush regulates many genes related to hemocyte function and/or differentiation also in S2 cells - some directly, some indirectly, some in cooperation with dMi-2, some independently of dMi-2. The additional data that we have generated at the reviewer's request further substantiates this claim.

*3. They find different classes of targets. Given the wealth of data from Drosophila S2 cells, they could compare with existing data sets, chromatin state maps, to see whether the classes are in different chromatin contexts.*

We thank the reviewer for her/his suggestion. We have now performed two types of analyses:

First, we have determined H3K4me3 and H3K27me3 levels across hemocyte-related, cell cycle and lipid metabolism genes that are regulated by Ush. All three gene classes have elevated H3K4me3 but do not display elevated H3K27me3 levels surrounding the TSS. We did not detect meaningful differences in the H3K4me3 and H3K27me3 profiles between the three gene classes. Moreover, we performed the same analysis for hemocyte-related, cell cycle and lipid metabolism genes (GO terms R-DME-168249, 0022402/0010564 and 0016054/R-DME-556833/M00087, respectively) that were not regulated by Ush. These gene sets had H3K4me3 and H3K27me3 profiles that are very similar to the corresponding Ush-regulated gene sets. Taken together, we do not find appreciable differences in the chromatin context, as determined by H3K4me3 and H3K27me3 levels, between the three gene classes nor did we find differences in chromatin contexts that correlate with Ush regulation, at least at this level of analysis. We provide this data for the reviewer below but do not feel that the result is unexpected and that the manuscript would benefit from inclusion of these results.

Second, we have determined the levels of Ush occupancy across hemocyte-related, cell cycle and lipid metabolism genes that are regulated by Ush and across those genes that are not regulated by Ush. For all three gene classes we find a more pronounced Ush occupancy around the TSS for Ush-regulated genes compared to genes that are not regulated by Ush. This further supports our conclusion that a substantial fraction of Ush regulated genes are bound by Ush and, therefore, putative direct targets. We note again, that we do not claim that all Ush-regulated genes are direct targets. This data supports the data presented in Figure 2B but we do not feel that it adds much new information. Therefore, we provide this data for the reviewer below but, again, would prefer to not include it in the manuscript.

Although not specifically requested by this reviewer, we now have added a comparative analysis of histone marks (H3K4me1, H3K4me3, H3K27me3) at genes bound by Ush alone, by dMi-2 alone and bound by both factors to Figure 5 (new Figure 5E) as this has revealed interesting differences (discussed in the manuscript text).

*4. What approach have they taken for comparing the ChIP seq with the RNA seq data? It is not straightforward to link ChIP peaks to genes for this type of analysis.*

The reviewer is correct in pointing out that it is not straightforward to correlate RNA-seq and ChIP-seq data. To analyse data generated with mammalian cells a typical approach is to link each peak to the nearest gene using tools such as GREAT (which, however, cannot be used for *Drosophila*; http://great.stanford.edu/public/html/). This “nearest gene” approach is not optimal but is generally considered to be sufficiently accurate for most purposes.

In contrast to mammalian genomes, most genes are very close to each other in the *Drosophila* genome. Moreover, intergenic regions are generally smaller than intragenic regions (see also Figure 2B). Thus, most enhancers are located within gene bodies. We therefore defined a Ush-bound gene as a gene with at least one Ush ChIP-seq peak in its gene body or promoter. We are confident, that this approach will correctly link most Ush peaks to their designated targets.

*As S2 cells are not diploid, there is potential for some anomalies in the chromatin data due to variations in ploidy. How have the authors controlled for this in their analysis of the ChIP data?*

For peak calling we used the MACS2 algorithm that compares ChIP-seq signals with the corresponding input control signals. This removes potential variations introduced by ploidy.

*5. Can they justify using 1bp overlap for the comparison of U-shaped and NuRD binding profiles. If U-shaped is involved in recruiting NuRD the peaks should overlap more significantly (as suggested by the plots). Is the overlap statistically significant? Some of the other S2 ChIP data could be used as comparisons, to demonstrate that the overlaps are specific.*

The reviewer brings up an important point. Indeed, determining “co-localisation” of two factors based on ChIP-seq data is far from trivial and many different scenarios have to be considered.

For example, it is possible that a narrow peak of factor 1 is completely included within a broader peak of factor 2. If one would require a certain percentage of both peaks to overlap (e.g. > 50% for both factors), this obvious co-localisation would have to be excluded, because while 100% of the factor 1 peak overlaps with the factor 2 peak, less than 50% of the factor 2 peak overlaps with the factor 1 peak (see scheme below). Additional complications are generated if a peak of factor 1 overlaps with two peaks of factor 2 (see scheme below).

To avoid exclusion of relevant co-localisations, a minimal overlap of 1 bp is widely used (see also the original paper introducing the MACS algorithm we have used (Zhang et al., 2008, Figure 3A). Nevertheless, the reviewer’s concerns about the extent and significance of overlaps (given the minimal 1-bp-overlap threshold that we have applied) is very relevant. To address this point we have quantified the extent of peak overlaps by plotting the number of peaks vs. overlap size (using 50 bp bins) – see figure below. This analysis identifies median and average peak overlap sizes of 371 bp and 469 bp, respectively. This demonstrates that the high degree of Ush and dMi-2 co-localisation that we have identified is not a consequence of using the low “1-bp-overlap” threshold.

*If U-shaped is involved in recruiting NuRD the peaks should overlap more significantly (as suggested by the plots).*

First of all, it is important to note that we do not claim that U-shaped is essential for dNuRD recruitment. We and others have shown that multiple proteins and nucleic acids are involved in recruitment of NuRD to chromatin, both in *Drosophila* and in mammals. In addition, NuRD possesses intrinsic DNA and histone binding activities which are believed to also contribute to chromatin association. Therefore, even if Ush was actively recruiting NuRD to chromatin (which we do not claim) the extent of Ush/dMi-2 co-localisation would be very much influenced by the prevalence of other recruiting factors and recruitment mechanisms.

*Is the overlap statistically significant?*

We have used a hypergeometric probability test to determine the p-value of the overlap of Ush and dMi-2-bound genes. This p-value is 1x10^-3000^ which is a meaningless number but does clearly demonstrate that the overlap is statistically significant.

*6. Controls with depletion of Ush should be included for the hhF41 enhancer in Figure 7.*

The effects of gain and of loss of Ush function on hhF4f enhancer activity in the lymph gland are well documented in the literature (Tokusumi *et al.*, 2010). Nevertheless, we now include additional controls confirming derepression of hhF4f enhancer activity in medullary and cortical zones of the lymph gland both in ush mutant lines and in lines where Ush expression is depleted by RNAi (new Supplementary Figure 7).

*What are the numbers (N) for the data in Figure 7H.*

The numbers (N) are given in Supplementary Table 2 which accompanies Figure 7H. This table contains detailed descriptions of all crosses analysed in our study including the numbers (N) of larvae tested for each cross.

*7. More information should be provided about the CRISPR engineering in the S2 cells. What were the “minor changes” what were the sequences used for targeting and how were the correctly engineered cells selected?*

We have now included a more detailed description of the CRISPR-mediated generation of S2 cell lines as well as the sequences used for targeting (see Methods section). In addition, we have included schematic outlines of the procedure in the new Supplementary Figures 1A (for Ush tagging) and 1C (for dMi-2 tagging).

**Reviewer #2**

We thank the reviewer for her/his comments. We appreciate the time an effort that went into many suggestions that aim to improve our manuscript.

*Lenz et al further dissect the function of Drospholia Ush in S2 cells and in vivo. They use proteomics, transcriptomics and genetic studies to show that Ush has multiple isoforms, one which interacts with the dNuRD complex and functions differently to the other isoforms. It is a nice study revealing an example of how a single protein can act in multiple mechanisms using alternative splicing.*

*Main comments:*

*1. It is currently unclear how many biological replicates were performed: please add this information into all figures, as well as providing an overview of the datasets generated, with the information about repeats and controls into the methods. This is particularly important for the RNAi experiments, where the authors need to show how efficient the knockdown was for each independent replicate, with the resultant overlap of DE genes between replicates.*

We have now added information about the number of biological replicates for each experiment that contains quantitative data and/or statistical analysis to the figure legends. We have also amended the methods section with an overview of all datasets and information about replicate number and controls:

“Raw and analysed data can be accessed at the GEO database: accession no. GSE146382.

In particular this includes raw reads from RNA-seq experiments (3 replicates each: dsEGFP, dsUsh, dsMi-2; 4 replicates each: dsEGFP, dsUsh-B) and ChIP-seq experiments (1 replicate each: Ush-GFP input, Ush-GFP ChIP, dMi-2-GFP input, dMi-2-GFP ChIP). For RNA-seq the following analyzed data are provided: Normalized counts (dsEGFP, dsUsh & dsMi-2 replicates), differentially expressed genes (dsEGFP vs. dsUsh; dsEGFP vs. dsMi-2), Normalized counts (dsEGFP & dsUsh-B replicates), differentially expressed genes (dsEGFP vs. dsUsh-B). Coverage tracks of Ush and dMi-2 ChIP-seq experiments as well as corresponding inputs are available for two different *Drosophila* genome versions (bigWig files for dm3 and dm6).”

We now provide a principle component analysis to estimate how comparable transcriptome changes are between corresponding replicates and to assess RNA-seq data quality. We demonstrate that corresponding triplicates cluster closely together and are highly concordant (see figure below). This confirms that knockdown generates reproducible transcriptome changes across all triplicates (see figure below).

Figure 1A demonstrates that the construct we have used for knockdown of Ush in the RNA-seq experiments is very efficient in depleting Ush from S2 cells. In this experiment we have used a Ush antibody provided by our co-author Prof. Robert Schulz early in 2019. We have run out of this antibody. Unfortunately, Prof. Schulz has passed away, his lab has been disbanded and despite our best efforts we have been unable to obtain additional Ush antibody. Therefore, we could not probe the RNA-seq samples for Ush knockdown. While the RNA-seq data does show decrease of Ush RNA, in our experience this does not reflect the extent of depletion of the protein and is a poor indicator of knockdown efficiency.

We note that the efficiency of knockdown of Ush and dMi-2 on the protein level is demonstrated multiple times in our manuscript (Figures 1A, 3E, 4D). Nevertheless, to provide further evidence that the RNAi constructs used in the RNA-seq experiments result in reproducible, efficient knockdown in S2 cells we provide the new Western Blot analysis below. This shows simultaneous knockdown of all Ush isoforms in S2 cells CRISPR-edited to express Ush-GFP (A) or Ush-FLAG (C) (using these lines circumvents the requirement of Ush antibody), specific knockdown of Ush-B ((B) and (D)) and knockdown of dMi-2 in S2 cells ((E); the three replicates used in the RNAseq experiment).

*2. Following on from the previous comment, the RNAi for Ush-b looks less effective than for total Ush (Figure 3D), which might be why you see less genes changing and no cell cycle arrest, rather than differences in isoform function.*

This is an astute observation by the reviewer. It is not easy to quantify relative knockdown (KD) efficiency of both isoforms from the Western Blot analysis in Figure 3D (Figure 3E in the revised manuscript). Nevertheless, we do see effective KD of Ush-B when we use an RNAi construct specific for Ush-B (at least, we can no longer detect a signal for Ush-B by Western Blot; see e.g. Figure 4D). Therefore, we think it is unlikely that the smaller number of genes responding to Ush-B KD (compared to the simultaneous KD of all isoforms) is caused by inefficient Ush-B depletion. We favour the hypothesis that Ush-A and Ush-B repress many genes in a redundant fashion. As a consequence only genes the repression of which is particularly dependent on Ush-B respond to Ush-B-specific KD. This is discussed on page 18 (‘*Impact of Ush-B/dNuRD on transcription in S2 cells”*) of the revised manuscript.

*3. Please include more information as to how the ChIP peaks are annotated as within Galaxy/Cistrome different regions can be set.*

We used MACS2 analysis under standard setting, specifically:

Set lower mfold bound = 5

Set upper mfold bound = 50

Band width for picking regions to compute fragment size = 300

Peak detection based on = q-value

Minimum FDR = 0.05

Effective Genome Size: Drosophila Genome

We have also added this information to the Methods section:

“Peaks were identified using MACS2 (Zhang et al., 2008) with the following settings: Set lower mfold bound = 5; Set upper mfold bound = 50; Band width for picking regions to compute fragment size = 300; Peak detection based on = q-value; Minimum FDR = 0.05. Overlap between peaks was obtained using the Venn Diagram tool within Galaxy/Cistrome platform. Peaks were considered overlapping at ≥ 1 common nucleotide.”

*How was a promoter peak or an enhancer peak annotated and how was an enhancer peak linked to a gene for comparing the ChIP-seq with DE genes to generate your direct transcriptional targets of Ush.*

We did not annotate enhancer peaks. Promoter peaks were defined based on their distance to annotated transcriptional start sites (+/- 1000 bp).

As to how ChIP-seq peaks were linked to DE genes we would like to repeat our answer to Reviewer #1 who has asked a very similar question:

The reviewer is correct in pointing out that it is not straightforward to correlate RNA-seq and ChIP-seq data. To analyse data generated with mammalian cells a typical approach is to link each peak to the nearest gene using tools such as GREAT (which, however, cannot be used for *Drosophila*; http://great.stanford.edu/public/html/). This “nearest gene” approach is not optimal but is generally considered to be sufficiently accurate for most purposes.

In contrast to mammalian genomes, most genes are very close to each other in the *Drosophila* genome. Moreover, intergenic regions are generally smaller than intragenic regions (see also Figure 2B). Thus, most enhancers are located within gene bodies. We therefore defined a Ush-bound gene as a gene with at least one Ush ChIP-seq peak in its gene body or promoter. We are confident, that this approach will correctly link most Ush peaks to their designated targets.

*4. The genes selected in Figure 2E, are these bound by Ush as well as misregulated after dsUsh? If yes, please make that clear in the text, if not Figure 2C-E should be complemented by the same plots but only using direct targets.*

*a. Likewise, please make clear whether the genes in Figure 6 are bound by Ush and by Mi-2 from your ChIP analyses.*

Please, find below a table listing whether the genes analysed in Figure 2E and Figure 6 contain a Ush or dMi-2 ChIP-seq peak within their promotor and/or gene body. Note that following a suggestion from reviewer #1 we have added two genes (lz and attila) and removed two others (Gs2, Ugt36Bc – greyed out) from the analysis. We have deliberately selected genes bound by Ush as well as genes not bound by Ush as Ush affects gene regulation both directly and indirectly (see Figure 2B). We do not think that direct regulation by Ush is necessarily more relevant than indirect regulation for establishing and/or maintaining specific gene expression programmes. Nevertheless, we now state in the text which genes are bound by Ush and which genes are not. We thank the reviewer for her/his comment as this will avoid potential confusion.

“We selected representative genes from each class to confirm their regulation by Ush by RT-qPCR following Ush depletion (**Figure 2E**). Some of these genes had Ush ChIP-seq peaks within gene body and/or promoter and are, therefore, putative direct targets (CG16267, pirk, GILT3, Lozenge, CHES-1, Cyclin B, Mcad, Echs1, ACC, fa2h). Others were not bound by Ush and represent genes that might be indirectly regulated by Ush (Attila, AurB, CDK1, polo, CROT).”

|  |  | annotated peak within promoter and/or gene body | |
| --- | --- | --- | --- |
| Genes validated by RTqPCR | category | Ush-GFP | dMi-2-GFP |
| CG16267 | hemocyte-related | yes | yes |
| Gs2 | hemocyte-related | yes | yes |
| pirk | hemocyte-related | yes | yes |
| GILT3 | hemocyte-related | yes | no |
| Ugt36Bc | hemocyte-related | yes | no |
| Lozenge | hemocyte-related | yes | no |
| atilla | hemocyte-related | no | no |
| AurB | cell cycle | no | no |
| CHES-1 | cell cycle | yes | yes |
| CDK1 | cell cycle | no | yes |
| polo | cell cycle | no | yes |
| Cyclin B | cell cycle | yes | yes |
| Mcad | lipid metabolism | yes | yes |
| Echs1 | lipid metabolism | yes | yes |
| ACC | lipid metabolism | yes | yes |
| CROT | lipid metabolism | no | no |
| fa2h | lipid metabolism | yes | yes |

*5. Please provide a schematic of the knock-ins of dUsh and dMi-2 including what was knocked in and where in the genes (adding the location of the primers used in Supp Fig1 would be helpful). Plus, it is important to show that these knock-ins did not create a phenotype and alter the normal function of dUsh and dMi-2.*

We have now included a detailed description of the CRISPR-mediated generation of S2 cell lines as well as the sequences used for targeting (Methods section “Endogenous tagging using CRISPR/Cas9”, too long to reproduce here). In addition, we have included schematic outlines of the procedure in the new Supplementary Figures 1A (for Ush tagging) and 1C (for dMi-2 tagging).

The knock-in did not display any discernable phenotype with respect to morphology, viability or proliferation. Adding a C-terminal tag to Ush did not affect its nuclear localisation or its ability to interact with NuRD (Figure 4C, 4I). Likewise, epitope-tagged dMi-2 still formed the NuRD complex and bound to Ush (Figure 4D). Moreover, the ChIP-seq data set obtained with epitope-tagged dMi-2 in this study is highly similar to two ChIP-seq data sets generated previously with two independent dMi-2 antibodies (Supplementary Figure 4) confirming that chromatin association was not significantly affected by the knock-in.

*6. S2 cells seem to be the perfect in vitro system for this work as it is a hemocyte-derived cell line. However, how much do they re-capitulate normal fate choices? As the authors mention, changes in Ush are associated with cell fate when pro-hemocytes differentiate to to plasmatocytes, crystal cells and lamellocyte. When you knock-down Ush, and many hemocyte-related genes change, does this result in a change in cell fate for the S2 cells? Likewise, with the knockdown of Mi-2, what is the phenotype in this cell-line?*

While S2 cells recapitulate many aspects of hemocytes (general gene expression profile, phagocytosis) they do not differentiate into crystal cells or plasmatocytes after Ush KD (based on the absence of the dramatic morphological changes that would be expected; if this would work it would be fantastic!). We think that while Ush KD steers the gene expression profile into the direction of crystal cells (e.g. by derepressing the crystal cell master regulator Lz) and lamellocytes (e.g. by derepressing Attila – see Figure 2E) this is not sufficient for full differentiation. Indeed, in the larval lymph gland, differentiation requires a complex interplay between the different cell populations present which engages numerous signalling pathways and is carefully orchestrated in both space and time. In essence, we believe that differentiation signals that pro-hemocytes receive in the lymph gland are absent in the S2 cell culture system.

dMi-2 KD likewise does not result in differentiation of S2 cells. Also, dMi-2 cooperates with many transcriptional regulators in addition to Ush and any effects of dMi-2 KD are likely to be more general. Indeed, we observe a gradual loss of viability after prolonged dMi-2 KD (7 days plus) suggesting that it has essential functions. However, we observe no significant reduction of viability under the KD conditions used in this study (the same is true for Ush and dMTA KD). We have added a viability assay to verify that none of the RNAi treatments adversely affects cell survival (new Supplementary Figure 6B).

*7. The transcriptomic and proteomic analyses were assessed after 3 or 4 days after RNAi and dsUsh transfected cells do not show normal proliferation after 24 hours. What is the viability of the cells after 3 or 4 days?*

See above. RNAi treatment does not reduce cell viability (new Figure 3B and new Supplementary Figure 6B). This is an important point and we are glad that the reviewer’s comment has prompted us to demonstrate more rigorously that RNAi effects on transcription or cell cycle are not a consequence of reduced cell viability.

*8. Please provide details of whether your differentially expressed genes after dsMi-2 is similar to previous reports.*

We have found one publicly available dataset for changes to the S2 transcriptome after Mi-2 knockdown (Reddy et al., 2010; PMID: 20733004). This data was generated using microarray technology. Despite the substantial difference in methodology we find a highly significant, positive correlation between the two data sets (see Figure below). Importantly, the expression of key hemocyte-related genes (see Figure below and Figure 6C), were similarly in the microarray experiment.

| hemocyte-related gene | FC RNA-seq (this study) | FC Microarray (Reddy et al. 2010) |
| --- | --- | --- |
| CG16762 | 2,954 | 1,121 |
| Lz | 1,279 | 0,959 |
| pirk | 3,038 | 4,162 |
| GILT3 | 2,711 | 1,955 |
| atilla | 1,098 | 0,991 |

*Minor points:*

*1. As Ush-wt peptide can less efficiently replace GST-mFOG1 than FOG1-wt even though both sequences have the exact same FOG repression motif implies the binding is not completely due to this motif. In addition, the sizes of the Ush peptides seen (180 and 220KDa) do not reflect the size of the predicted proteins (Ush-b is predicted to be 125kDa, Ush-a/c 127kDA, Ush-d 122kDA). This implies there are post-translational modifications, which might play a part in how Ush interacts with NuRD. Therefore, while we agree that the N-terminus of Ush is critical for binding, I would suggest discussing this in the discussion.*

The reviewer makes a very interesting point. We have added the following paragraph to the discussion:

“It is interesting that the FOG1 peptide used in our study binds with higher affinity to dNuRD than the Ush peptide even though the FOG repression motif contained within both peptides is identical. This suggests that amino acids outside of the FOG repression motif contribute to dNuRD binding. It is also possible that interaction between dNuRD and Ush is modulated by post-translational modification within or in the vicinity of the FOG repression motif *in vivo*. Indeed, phosphorylation of serine residue 2 within the FOG repression motif has previously been shown to lower NuRD binding (Dubuissez et al, 2016).”

We also agree with the reviewer that phosphorylation might account for the discrepancy of the theoretical molecular mass of Ush isoforms and their apparent molecular mass as determined by SDS-PAGE. However, we wonder if one (or a few) phosphorylation sites would be able to account for such a size difference (50-100 kDa). We speculate that amino acid composition and/or the isoelectric point might also influence electrophoretic mobility. For example, stretches of negatively charged amino acids can significantly slow migration during SDS PAGE (presumably, because these regions bind less SDS). We have demonstrated this for dMEP1 which has a theoretical MW of 124 kDa yet migrates with an apparent molecular mass of 180 kDa (see Figure 4C). Here, deletion of the D/E stretch “normalises” electrophoretic mobility (*data not shown*). Since we have no data shedding light on the unexpected electrophoretic mobility of Ush proteins we would prefer not to speculate about this in the manuscript.

*2. “However, our findings also suggest that Ush forms complexes with bHLH transcription factors” in the first results section should be toned down. You do not show Co-IPs for Ush and bHLH TFs, but show that they bind to the same locations.*

Agreed. We have toned down the statement as follows:

“However, our findings also hint towards a possible complex formation with bHLH transcription factors, GAGA factor and general transcription factors binding to the initiator element.”

*3. A more in depth comparison of what is known about FOG1 and Ush roles in haematopoiesis in the introduction should be included to understand better the potentially conserved roles.*

We do not feel that an “in depth” comparison between FOG1 and Ush functions in hematopoiesis in the introduction is necessary. However, we do compare the roles of both proteins in the discussion:

“Although the process of hematopoiesis in *Drosophila* is far less complex than in mammals, Ush and FOG1 play remarkably similar roles in suppressing certain hematopoietic lineages. FOG1 facilitates erythroid and megakaryocyte differentiation while suppressing mast cell differentiation. While high Ush levels in hemocyte progenitors counteracts differentiation into all three *Drosophila* hemocyte cell types, intermediate Ush levels are sufficient to suppress crystal cell and lamellocyte differentiation but compatible with differentiation of plasmatocytes (Fossett, 2013). Both FOG1 and Ush cooperate with NuRD using a highly conserved short peptide motif. Thus, our study identifies the FOG1/Ush-NuRD complex as an ancient component of the machinery regulating hematopoiesis.”

We think that this sufficiently highlights the conserved functions of both proteins without going into too much detail.

*4. In “Ush-B/dNuRD complex does not regulate the cell cycle” results section, “…..depletion of Ush-B, dMi-2 or dMTA1-like did not alter protein expression levels of Cyclin A or Cyclin B (Figure 3C)” should say Figure 3D.*

Thank you. We have corrected this.

*5. Ush hypomorph and null allele names are in very small font and cannot be read in the “Ush/dNuRD regulate hemocyte differentiation in vivo” results section as well as figure legends.*

The text uses normal font sizes, the labeling in the figure has been enlarged.

**Reviewer #3**

We thank the reviewer for his insightful, helpful and entertaining comments and suggestions.

*In this manuscript, the authors use biochemical and cell biological approaches to examine the function of the Drosophila transcriptional regulator Ush.*

*The authors first carry out ChIPseq on endogenously tagged Ush in S2 cells. They find it binds to 7K sites that are enriched for promoters – and that the sites are are enriched for GATA sites (and other TF binding sites).*

*Given that they have conducted a ChIPseq analysis of Ush, the authors should make some comment on whether the protein is likely to bind DNA. In so doing, they could refer to existing data on FOG1.*

We thank the reviewer for her/his suggestion. Indeed, direct DNA binding by FOG1/Ush has to our knowledge not been reported and it is widely believed that association with DNA/chromatin requires binding to GATA-type transcription factors. To make this clear, we have added the statement

“Ush and FOG1 do not bind DNA directly and are recruited to their sites of action by sequence-specific GATA transcription factors.”

to the introduction.

*Next, they use RNAi to deplete Ush and show by RNAseq that 1200 transcripts are upregulated and 600 downregulated. They note that a significant number of these genes are bound by Ush, suggesting that they might be direct Ush targets. Perhaps the authors could indicate in the text what they mean by a gene being bound by USh. Do they mean promoter+gene body? Or something else? Given that we don’t really have very good data on which enhancers are associated with which genes, perhaps their calculated numbers are underestimates, even?*

These are all very good points. Indeed, our definition of “Ush bound gene” is a minimum of one ChIP-seq peak in the promoter and/or gene body. To make this clear we have now changed the relevant section to:

“Comparison of the RNA-seq and ChIP-seq datasets revealed that approximately half of Ush-repressed genes (651 of 1268, 51%) and one third of Ush-activated genes (175 of 560, 31%) contain a Ush ChIP-seq peak in the promoter or gene body (**Figure 2B**). These 826 genes are, therefore, likely to be direct transcriptional targets of Ush.”

With regard to enhancers we would like to reiterate a response to reviewer #1 here:

“In contrast to mammalian genomes, most genes are very close to each other in the Drosophila genome. Moreover, intergenic regions are generally smaller than intragenic regions (see also Figure 2B). Thus, most enhancers are located within gene bodies. We therefore defined a Ush-bound gene as a gene with at least one Ush ChIPseq peak in its gene body or promoter. We are confident, that this approach will correctly link most Ush peaks to their designated targets.”

Nevertheless, it is still possible that Ush binds to distally located enhancers and that we fail to assign this ChIP-seq peak to its proper target gene. The reviewer is right in stating that we might be underestimating the number of direct targets to some extent.

*Next they conduct a gene ontology analysis of the genes that were altered in expression under RNAi. Perhaps it would be good if instead or as well they used the set of USh genes that they estimated to be direct targets by ChIPseq. Does this change the ontology analysis?*

We have now performed Gene Ontology analysis on Ush-regulated genes that are also bound by Ush. The top 100 hits are shown below. Also in this subset of Ush-regulated genes we find GO terms that can be categorized as “hemocyte-related” (green), “cell cycle” (yellow) and “lipid metabolism” (blue) genes. This further substantiates our finding that Ush influences the expression of genes relevant for these biological processes. As we are only looking at a subset of Ush-dependent genes here, the abundance and relative distribution of characteristic terms differs from our original analysis in Fig 2C. This is to be expected.

Biologically relevant changes upon Ush knockdown are likely a combination of changes in expression of both Ush-bound genes (direct targets) and genes that are not bound by Ush (indirect targets). Therefore, we believe that a GO term analysis of all Ush-regulated genes (as shown in Figure 2C) is more appropriate.

*The authors note that hemocyte-related genes regulated by Ush are overwhelmingly repressed by Ush as are lipid metabolism genes. The opposite is true for cell cycle related genes. This seems an interesting finding.*

*RT-qPCR was carried out to corroborate the RNAseq data. The effect sizes are large for the hemocyte- and lipid metabolism-related genes. However, the effect sizes are quite small for the cell cycle genes – all less than two-fold (though the authors do show an effect on cell cycle subsequently). The authors mark these results as statistically significant. I have a couple of issues with this presentation. One is that the figure legend says that the error bars represent the SD from technical triplicates. The authors should be aware that technical replicates cannot be used for drawing statistical inferences. Either they have just written the wrong thing in the legend (and their replicates are *biological* replicates) or they need to remove all the error bars shown and remove the T test calculations. Second, even if their data really do represent triplicate independent measurements (and don’t get me started about the independence of replicates in most cell biology experiments…), the less-than-two-fold effect sizes for the cell cycle genes would lead me to conclude that it’s quite unlikely that there is a real/interesting effect here (based on these data alone). For one thing, I would have thought it’s quite hard, given the nature of a qPCR experiment, to be confident about a two-fold effect – the experiment involves measuring midpoints that arise from PCR cycles (doublings), so it feels like you’re pushing the limits of the technique to try to draw inferences about two-fold (or smaller) effects, unless you have true biological replicates (which cell biology experiments often don’t – different aliquots of the same cells from the freezer etc). As a corollary, the authors should note that there is a move in the literature to showing actual data points on bar graphs rather than just the bars. In this case, if their data points are technical replicates, then they only have one data point to show for each bar (the mean of the technical triplicates).*

The reviewer makes several good points. We now display the data in Figure 2E showing individual data points for five biological replicates. From these we have derived standard deviations and determined the level of significance using a paired, two-tailed Student’s T-test.

We have amended the information about the number of biological replicates and statistic analysis in all figure legends.

We respectfully disagree with the reviewer’s assessment that a qPCR experiment is unable to reliably determine differences in the 2-fold range. We also would like to point out that the 2-fold decreases in the expression of cell cycle genes correlate with (a) clear proliferation and cell cycle defects (as noted by the reviewer below) and (b) decreased Cyclin A and Cyclin B protein levels (Figure 3E). We think this suggests that the mild changes in cyclin gene transcription are indeed physiologically relevant.

*Finally, these comments also apply to Figure 6 (in particular Fig 6C, which talks about technical triplicates). Really, the world would be a better place in most cases without these t-tests – given how unstable p values are for small sample sizes and small effect sizes (and therefore how little confidence can be attached to them) – and how arbitrary the 0.05 cutoff is, but that’s a battle for another day.*

*That was a bit of a rave, but I think there is *something* in there for the authors to address… and either way it doesn’t significantly affect the main thrust of the manuscript.*

We thank the reviewer for the “rave” as it has led to a stimulating discussion about how to present our data. We now have also changed Figure 6C to display individual data points of 5 biological replicates from which we derive error bars and statistical significance as in Figure 2E.

*Flying in the face of the questions I have about the qPCR data, the authors go on to show an effect of Ush knockdown on cell cycle.*

*The authors next examine the splice isoforms of Ush, to ask whether activation vs repression functions of Ush might be associated with these isoforms. Interestingly, they show that only one of the isoforms (UshB) contains an N-terminal sequence that is already known from published work on FOG1 to interact with the NuRD complex. They nicely demonstrate that this is the case for Ush – only UshB interacts with NuRD components and the interaction is clearly mediated by the N-terminal motif identified previously in other proteins such as FOG1.*

We thank the author for these nice comments.

*I would just note in this section that the authors say things such as “We conclude that dMi-2 specifically interacts with the Ush-B isoform”. However, the nature of a coIP is that you cannot identify direct interactions – only conclude that two proteins can exist in the same complex. Many people would read “specifically interacts” as meaning a direct interaction, which is a bit misleading I think (unintentionally here, I have no doubt).*

We certainly do not imply a direct interaction when we use the term “specific interaction”. Frankly, it had not crossed our mind that this might be misinterpreted in this way. Nevertheless, we have changed this statement to:

“We conclude that dMi-2 specifically forms a complex with the Ush-B isoform.”

*The authors show that dMi2 and Ush ChIPseq datasets show a lot of peak overlap. It would be nice to see a breakdown of the overlapping vs non-overlapping peaks. Are they enriched/depleted for active/repressed genes compared to all dMi2/Ush peaks? What about promoters/enhancers etc? GATA sites? It feels like there is information in here that could be teased out.*

We have now separately assigned Ush-only, dMi-2-only and dUsh/dMi-2 (co-occupation) peaks to promoters, exons, introns and intergenic regions (see new Figure 5D). As discussed above we are not able to confidently assign peaks to enhancers but note again that, in the *Drosophila* genome, many enhancers lie within introns. We have also added a comparative analysis of histone marks (H3K4me1, H3K4me3, H3K27me3) at genes bound by Ush alone, by dMi-2 alone and bound by both factors (new Figure 5E). This led to interesting findings that further support our hypothesis. We have added the following section to describe these results:

“We then assigned dUsh/dMi-2 co-occupied regions as well as “Ush-only” and “dMi-2-only” ChIP-seq peaks to genomic regions (**Figure 5D**). Ush-only peaks show a strong preference of intron. dMi-2-only peaks on the other hand are most strongly enriched at promoters. Co-occupied regions show preferential association with both promoters and introns. In agreement with these findings analysis of histone marks at Ush-only peaks revealed a strong enrichment of H3K4me1, a histone modification that is characteristic for enhancers (**Figure 5E**). dMi-2-only peaks contained high levels of H3K4me3, a hallmark of active promoters. Co-occupied regions displayed elevated levels of both H3K4me1 and H3K4me3.”

*It would also be nice to know what is meant by the peaks ‘overlapping’. That would involve some assessment of peak width in the experiments. For example, if the peaks are 500 bp wide (I just made that number up), does overlap mean overlap of 20% of the peak area? 50%? Something else? Knowing that dMi2 and Ush were 500 bp apart is different from knowing they’re 20 bp apart on DNA.*

The reviewer brings up an important point. Indeed, determining “co-localisation” of two factors based on ChIP-seq data is far from trivial and many different scenarios have to be considered.

For example, it is possible that a narrow peak of factor 1 is completely included within a broader peak of factor 2. If one would require a certain percentage of both peaks to overlap (e.g. > 50% for both factors), this obvious co-localisation would have to be excluded, because while 100% of the factor 1 peak overlaps with the factor 2 peak, less than 50% of the factor 2 peak overlaps with the factor 1 peak (see scheme below). Additional complications are generated if a peak of factor 1 overlaps with two peaks of factor 2 (see scheme below).

To avoid exclusion of relevant co-localisations, a minimal overlap of 1 bp is widely used (see also the original paper introducing the MACS algorithm we have used (Zhang et al., 2008, Figure 3A). Nevertheless, the reviewer’s concerns about the extent and significance of overlaps (given the minimal 1-bp-overlap threshold that we have applied) is very relevant. To address this point we have quantified the extent of peak overlaps by plotting the number of peaks vs. overlap size (using 50 bp bins) – see figure below. This analysis identifies median and average peak overlap sizes of 371 bp and 469 bp, respectively. This demonstrates that the high degree of Ush and dMi-2 co-localisation that we have identified is not a consequence of using the low “1-bp-overlap” threshold.

*Next, the authors carry out RNAseq and RT-qPCR on S2 cells specifically depleted for UshB (and it’s very nice that they are able to do this) or for dMi2. They show that these treatments to not alter expression of cell cycle genes or (nearly all) lipid metabolism genes. They comment that genes involved in hemocyte development *are* affected, but they are a bit vague: “changes in several genes related to…”. Could they be more quantitative here please? And it would be good if they compared the genes that change in the UshB knockdown with those that change in the Ush knockdown. What is the overlap? In each functional class?*

The GO term analysis of transcriptional changes after dMi-2 knockdown has revealed the GO term “Innate Immune Response GO:0045087” as enriched (in contrast to mammals, who have both adaptive and innate immune systems, *Drosophila* only has an innate immune system which is to a large part embodied by the macrophages under investigation in our study). This GO term contains 91 genes that respond to dMi-2 knockdown. This demonstrates a strong impact of dMi-2 on genes related to macrophage function.

Ush-B specific depletion leads to comparatively few changes to the transcriptome (we have discussed possible reasons for this in the manuscript). In Supplementary Figure 5 and Supplementary Table 1 we list all genes (85 in total) differentially expressed after Ush-B knockdown. We also state in the manuscript that 18 of these (21%) are hemocyte-related which is a significant fraction. We have, of course, considered the overlap between Ush and Ush-B regulated genes and found that, e.g. 28 out of 47 genes that are upregulated upon Ush-B knockdown are also upregulated upon knockdown of all Ush isoforms. This demonstrates an (expected) large degree of overlap. However, given that the numbers of genes in the two sets (>1.000 for the total Ush knockdown; <100 for the Ush-B knockdown) are so different we do not consider such an analysis particularly meaningful.

*They go on to show that NuRD and Ush are involved in specific hematopoietic events. I won’t try to comment on the fly biology experiments as I am not an expert in this area.*

*In their discussion, the authors comment on the Tal1/LMO2/LDB1 (they need to correct the “LBD1” that they write) complex in mammals. It would be good for them to comment on the biochemistry of the fly versions of these proteins: namely Chip, LMO, DA/AC/SC. I thought there was evidence that these proteins come together with Pannier to form complexes – which makes it very likely that Ush is involved too. Are there ChIPseq data on these other proteins that could be compared to the authors’ Ush ChIPseq data?*

It is correct that interaction between CHIP and other Tal1/LMO2/LDB1 homologs have been reported although these data are mostly based on interactions detected in vitro or after overexpression of the putative interaction partners. Moreover, the proteomics data we used to uncover Ush interactors in S2 cells (Guruharsha et al., 2011) did not reveal Chip, LMO or DA/AC/SC as interactors of Ush at the biochemical level. In addition, while Pannier’s role in heart and neural development is well described in the literature, it does not seem to function during fly hematopoiesis. As the reviewer suspects it has indeed been demonstrated that Pannier cooperates with Ush. As these processes are unrelated to hematopoiesis (and our manuscript is very long as it is) we would prefer not to discuss this. To our knowledge there are no ChIP-seq data sets for these factors (at least in S2 cells).

*The authors also comment on the FOG repression motif and say that they’ve identified one in OAZ. It’s worth noting that this motif doesn’t have to be at the N-terminus of a protein to bind to NuRD (e.g., PHF6).*

This is correct.

*The really interesting question that this work raises – that the authors try to address in their discussion, but can’t really – is why UshB depletion only affects a functionally distinct set of Ush target genes out of the sets identified in their earlier Ush knockdown experiment (that is, the hemocyte ones). They suggest that the hemocyte genes might be particularly dependent on NuRD or high levels of Ush. This is possible but it’s hard at this stage to know why that might be. That’s OK – they can’t answer *every* question in this paper.*

We thank the reviewer for this comment and completely agree.

*In this regard, it would have been nice if the authors could have determined which sites are bound by UshB in the genome and which by non-NuRD binding isoforms. Might this not give some insight into whether UshB is distributed across all Ush sites or enriched at a subset. They *could* have done ChIPseq on their UshB-depleted cells to answer this question, couldn’t they? I don’t expect them to do that experiment in COVID world, but they could at least comment on it one way or the other perhaps.*

The main problem is that the antibodies we have available do not distinguish between different Ush isoforms. A Ush ChIPseq experiment after Ush-B depletion would have been a possibility. However, we do not think that Ush isoforms bind to distinct sites based on what we know about how they are recruited to chromatin. Ush does not possess sequence-specific DNA binding activity itself. It is directed towards chromatin via interaction with GATA-type transcription factors. The zinc fingers involved in mediating this interaction are shared by all Ush isoforms implying that they will all be recruited to the same sites. We hypothesize that the differences in Ush isoform function will manifest after recruitment, potentially by modulating NuRD activity (in the case of Ush-B).

*Overall, this manuscript is of high quality, well written and the story presented in a logical and straightforward way. The authors dissect Ush function in a clear and rigorous way, and are able to present several interesting conclusions that are of relevance to researchers trying to understand the mechanisms underlying gene regulation in eukaryotic organisms. Thus, I am of the view that this work is appropriate for publication in PLoS Genetics.*

We thank the reviewer for this assessment.

*Minor points*

*In the Introduction, the authors state:*

*“Changes in Ush levels govern cell fate choice: The stem cell-like pro-hemocytes express high levels of Ush. Ush expression is downregulated to lower levels as pro-hemocytes differentiate into plasmatocytes and crystal cells and completely shut off during lamellocyte differentiation (Fossett, 2013).”*

*However, their statement that Ush governs cell fate choice can’t be inferred from the following statements – one can only infer that Ush expression and hemocyte type are correlated. If it is known that the relationship is causal, ,the authors should instead mention data that make this connection.*

While we agree with the reviewer that correlation does not imply causation the references cited do indeed summarise studies demonstrating that experimentally changing the levels of functional Ush does indeed alter differentiation.

*“Established roll” should be “Established role”*

Thanks. Fixed.
