# Supplementary material for: Ush regulates hemocyte-specific gene expression, fatty acid metabolism and cell cycle progression and cooperates with dNuRD to orchestrate hematopoiesis
Source: PLoS Genet. 2021 Feb 18;17(2):e1009318. doi: 10.1371/journal.pgen.1009318 (PMC7891773; doi:10.1371/journal.pgen.1009318)
Supplement: Attachment — Submitted filename: Lenz et al Response to reviewers 2020-12-11.docx [file pgen.1009318.s014.docx]

**Rebuttal Letter**

(Reviewer comments in *Italics)*

Response to Reviewer #1's comments:

*The authors have performed additional analysis to clarify their results and have addressed many of the concerns raised. There remain some aspects that require further clarification.*

*First, to what extent are the S2 data representative of Ush role in the lymphgland. There are mixed messages about this. E.g. the answer to Reviewer 1 point 1 seems to suggest that depleting Ush has converse effects on proliferation in vivo. The discussion may be misleading on this point. And they need to be cautious about how much of the gene programmes can be extrapolated.*

We agree with the reviewer that our discussion does not adequately discuss the disparate effects of Ush loss of function on cell cycle, proliferation and differentiation in S2 cells and the lymph gland. We, therefore, have added two sentences to avoid potential confusion. The relevant paragraph now reads (added sentences in **bold**):

Ush regulates the cell cycle

Ush also regulates cell cycle genes in a coordinated fashion. The RNA levels of several genes encoding proteins essential for the entry into and progression through mitosis are maintained at appropriate levels by Ush. This transcriptional regulation has functional significance since Ush depleted S2 cells have strongly decreased proliferative capacity and exhibit a pronounced G2/M block. Ush is expressed in proliferating pro-hemocytes but downregulated in terminally differentiated plasmatocytes, crystal cells and lamellocytes (Fossett et al., 2001). We speculate that Ush supports the expansion of pro-hemocytes. Conversely, downregulation of Ush during lineage determination might allow these cells to exit the cell cycle for terminal differentiation. **While loss of Ush function does indeed result in an increase in the number of differentiated crystal cells in both embryos and larvae the predominant phenotype is hypertrophy of the larval lymph gland (Sorrentino et al., 2007; Fossett et al., 2001). These seemingly opposing effects suggest that Ush acts at multiple levels in the lymph gland not all of which are recapitulated in S2 cells.** The human Ush homolog FOG1 has also been proposed to play a pro-proliferative role when overexpressed in NIH 3T3 cells (Kadri et al., 2015; Kadri et al., 2009). However, this does not appear to involve the transcriptional regulation of cell cycle genes.

*Second, 2 concerns about the ChIP data. (i) There has only been one replicate performed. (ii) There is no GFP control - it’s possible that the ChIPs are detecting “open chromatin” that would overlap between the two samples. To some extent the concern re (i) is mitigated by the similarities to the existing Mi-2 RNA data. They could use the publicly available Mi-2 ChIP data from S2 cells and/or some of the other GFP ChIPs from S2 cells to discard these concerns.*

We offer four observations which argue against the hypothesis that sites co-occupied by Ush and dMi-2 are simply "open chromatin".

(1) Indeed, we have performed a GFP control in parallel (anti-GFP ChIPseq from the parental S2 cell line used for GFP tagging of Ush and dMi-2). This ChIP simply did not recover sufficient DNA for sequencing. In contrast to other antibodies (or IgG), the single chain GFP antibody used here does not bring down significant amounts of DNA. This argues against the hypothesis that this antibody unspecifically interacts with open chromatin. We had failed to mention this control in the manuscript but now have included the statement:

"As a control, we used the same antibody to precipitate chromatin from parental S2 cells expressing untagged Ush. This recovered only small amounts of DNA that were insufficient for sequencing."

(2) During the last two years we have used the same approach (CRISPR-mediated GFP tagging of endogenous proteins followed by anti-GFP ChIP-seq) to determine the genomewide binding profiles of several chromatin regulators in S2 cells (Macinkovic et al, NAR, 2019). This allows us to compare the Ush/dMi-2 chromatin binding profiles with those of other chromatin associated proteins. In general, the overlap between Ush and dMi-2 binding sites is far greater than between Ush/dMi-2 and any other chromatin associated proteins analysed by us. To illustrate this point we present a Venn diagramme and a gene browser screenshot comparing the chromatin binding profiles of dMi-2-GFP, Ush-GFP and dL(3)mbt-GFP. dL(3)mbt is a subunit of an abundant repressor complex (LINT) that is unrelated to Ush/dNuRD but likewise associated with a large number of genes. The overlap between dL(3)mbt binding sites and dMi-2 binding sites is substancial which is not surprising given that both dNuRD and LINT bind to many promoters. However, whereas 66% of Ush binding sites overlap with dMi-2 binding sites, only 30% of Ush binding sites overlap with dL(3)mbt binding sites. Thus, a large proportion of Ush/dMi-2 co-occupied sites is not bound by another chromatin-associated proteins.

3) As the reviewer notes, there is excellent concordance between the chromatin binding profile of dMi-2-GFP and those obtained with antibodies directed against dMi-2 (Supplementary Figure 4). If the majority of binding sites detected were due to an unspecific interaction of the GFP antibody with "open chromatin" two independent dMi-2 antibodies would have to be afflicted in a similar fashion. This is unlikely.

4) Ush binding sites are dramatically enriched for GATA factor binding sites. This makes perfect sense given the known role of GATA factors in the recruitment of Ush but cannot be explained if the signals obtained in our ChIP-seq experiment represent "open chromatin".

*Third, what is the significance of the interaction between Ush and NuRD? In the rebuttal (Reviewer 1 Point 5) they say “Therefore, even if Ush was actively recruiting NuRD to chromatin (which we do not claim)” If Ush is not recruiting NuRD, what is the role of the direct interactions they have detected? This is a major element of the paper so they need to make some explanation about the significance of these interactions. Indeed in the abstract they refer to “the Ush/NuRD complex”. What does Ush contribute if not helping to direct NuRD to specific targets?*

Our study identifies a robust physical interaction between Ush and dNuRD (which is why we refer to the Ush/NuRD "complex") as well as a functional cooperation between Ush and dMi-2/dNuRD in the regulation of transcription and differentiation. Our study does not characterise the molecular mechanism of this cooperation. The reviewer seems to be somewhat disappointed that we do not suggest that Ush recruits dNuRD to chromatin. While we acknowledge that this a popular model to describe the relationship between NuRD and FOG repression motif containing transcription factors in mammalian cells, we note that there is little evidence to support the notion that the recruitment model is generally applicable. In fact, it is clear that robust interactions between NuRD and FOG repression motif containing transcription factors, such as Sall4, exist even though Sall4 does not mediate NuRD chromatin associations (Miller et al, Development, 2016). Indeed, we have no data that would support the view that Ush recruits dNuRD to chromatin. Physical interactions between transcription factors and chromatin remodelers can have other consequences. For example, we have previously demonstrated that binding of the ecdysone receptor to dMi-2 activates its nucleosome remodeling activity (Kreher et al, Nature Comms, 2017). However, we consider this unlikely given that the FOG repression motif contacts the p55/RbAp48 and dMTA/MTA1/2/3 subsunits of NuRD rather than the CHD4/dMi-2 ATPase subunit. It is possible that the interaction of Ush with p55 and/or dMTA modulates the activity of the HDAC module of dNuRD but, again, we have no data to support this. In summary, we feel that the molecular mechanism of the Ush/dNuRD cooperation is beyond the scope of our study and we would prefer not to speculate on this. The latter would require a thorough and critical evaluation of the recruitment model. This would add more text to the discussion that the reviewer already feels is too long.

*Minor Points:*

*Reviewer 2 point 4:*

*The table is a valuable addition to the paper and helps the understanding, I suggest it is included.*

We thank the reviewer for this suggestion and have included the table as a new **Supplementary Table 1**

*The discussion is very long and could be significantly condensed.*

We agree that the discussion is long. However, we feel that this is warranted given the complexity of our results. Since the other reviewers have not raised the same objection we have decided to not shorten the discussion.

Response to Reviewer #2's comments:

*The authors have addressed all my comments well.*

*However, I was surprised to find there was only one replicate of the ChIP-seq experiments performed. While the authors compare the Mi-2 ChIP with two other data sets, the ChIP-seq for Ush is not- presumably because there are no published data sets already. There is high variability between ChIP experiments, and as such repeats would be beneficial to generate high confidence binding sites. While I am not asking for repeats to be performed, I would ask that a sentence be included somewhere in the manuscript to alert the reader that these sites maybe not be a comprehensive list of Ush binding sites due to only have one repeat.*

In principle, we agree with the reviewer. However, the variability between ChIP-seq experiments is generally lower than between (e.g.) RNA-seq experiments. Also, we refer the reader to our response to reviewer #1 above. The reviewer acknowledges that the dMi-2 ChIP-seq dataset is corroborated by two independent dMi-2 ChIP-seq data sets (Supplementary Figure 4) suggesting that our ChIP-seq approach (ChIP of GFP-tagged proteins with a single chain GFP antibody) is not systematically inferior to conventional ChIP-seq approaches. We are routinely using this strategy (see Macinkovic et al, NAR, 2019) and generally see a very high concordance between our datasets and published ChIP-seq datasets (another example would be the comparison of our Lint1-GFP with published Lint1 ChIP-seq datasets (Macinkovic et al, NAR, 2019)). This makes us confident that our Ush ChIPseq data set has a high chance of being comprehensive even though it is derived from only one replicate.

Having said all this, the reviewer is, of course, correct in stating that we cannot be sure and that we should alert the reader to this issue. Therefore, we have added the following sentence to the Bioinformatical Analysis section in the Methods:

"Given that ChIP-Seq data was generated from single replicates it should be noted that the peaks identified might not represent a complete list of all dMi-2/Ush binding sites."
